# A Modified Method Incorporating Multiplex PCR Reveals *Fusobacterium* Prevalence in Southern Chinese Population and Its Correlations in Cancers

**DOI:** 10.1111/1751-7915.70292

**Published:** 2025-12-25

**Authors:** Tingting Shen, Jiarui Liang, Xuyu Li, Xiaoxie Xu, Liqiong Li, Yinjuan Xu, Shanshui Zeng, Bingyu Li, Hui Li, Mengyao Hu, Lang Zhou, Siqi Yan, Ya Zhang, Ziwei Zhou, Huaaishi Liang, Shulei Chen, Zhikun Liang, Congrong Wang, Hongwei Zhou, Dongxin Zhang

**Affiliations:** ^1^ Microbiome Medicine Center, Department of Laboratory Medicine Zhujiang Hospital, Southern Medical University Guangzhou People's Republic of China; ^2^ Department of Laboratory Medicine Nanfang Hospital, Southern Medical University Guangzhou People's Republic of China; ^3^ Research Institute, DAAN Gene Co., Ltd. Guangzhou People's Republic of China; ^4^ Department of Chest Surgery Zhujiang Hospital, Southern Medical University Guangzhou People's Republic of China; ^5^ Guangzhou Dayuanqi Biotechnology Co., Ltd. Guangzhou People's Republic of China; ^6^ Guangdong Provincial Clinical Research Center for Laboratory Medicine Guangzhou People's Republic of China

**Keywords:** cancer, diagnosis, *Fusobacterium*, identification, multiplex PCR assay, population, prevalence

## Abstract

*Fusobacterium*, a gram‐negative anaerobic bacillus in mouth, gastrointestinal tract and elsewhere, has long been considered as opportunistic pathogen. Increasing evidence indicate the association of *Fusobacterium* with human diseases, especially cancer. However, previous studies demonstrated contradictory prevalent features of *Fusobacterium* species in normal and patient population. To address this dissonance, we developed a high‐precision multiplex PCR assay that allows concurrent identification of five species of *Fusobacterium* ( *F. nucleatum* , *F. mortiferum* , *F. ulcerans* , *F. varium* and *F. necrophorum* ) and four subspecies of *F. nucleatum* (*nucleatum*, *animalis*, *vincentii* and *polymorphum*). By employing the PCR method, we investigated the prevalent features of *Fusobacterium* communities in Southern Chinese population and cancer patients. Surprisingly, we found *F. nucleatum* was dominant in both Southern Chinese population and cancer patients, and discovered the correlations of *Fusobacterium* species to host conditions. Moreover, *F. mortiferum* exhibited better diagnostic performance for cancers compared to other species, and the combination of *F. mortiferum* , *F. nucleatum* , body mass index and haemoglobin by a logistic regression model showed excellent diagnostic performances for cancers. Additionally, we determined the compositional features and loads of *Fusobacterium* communities in paired tumour, adjacent tissues and normal tissues of colorectal cancer and lung cancer. Hence, we developed a high‐precision multiplex PCR assay to profile *Fusobacterium* in human faeces and tumour, and demonstrate its prevalence in Southern Chinese population with correlations to host conditions and cancers.

## Graphical abstract

By large‐scale comparative genomics, we identified species and subspecies‐specific genetic markers in *Fusobacterium*, and developed a high‐precision multiplex PCR assay that allows concurrent identification of five species of *Fusobacterium* ( *F. nucleatum* , *F. mortiferum* , *F. ulcerans* , *F. varium* and *F. necrophorum* ) and four subspecies of *F. nucleatum* (*nucleatum*, *animalis*, *vincentii* and *polymorphum*).

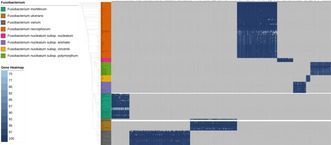

## 1 Introduction

*Fusobacterium*, a gram‐negative anaerobic bacillus found in human mouth and gastrointestinal tract, has long been considered as opportunistic pathogen. Although well known as opportunistic pathogen, the role of *Fusobacterium* as a cancer‐causing member of the microbiota is emerging (Gaiser et al. 2019; Hieken et al. 2016; Nejman et al. 2020). Numerous studies have shown that *Fusobacterium nucleatum* ( *F. nucleatum* ) is more prevalent in both gut (Dai et al. 2018; Yu et al. 2017; Thomas et al. 2019; Wirbel et al. 2019) and tumour tissue (Nakatsu et al. 2015; Castellarin et al. 2012; Kostic et al. 2013) of colorectal cancer (CRC) patients compared to healthy individuals and non‐CRC patients, and plays an important role in CRC development (Bullman et al. 2017; Brennan and Garrett 2019; Wang and Fang 2023). In addition to CRC, *F. nucleatum* was also found to affect the progression, prognosis and therapeutic response of other tumours. *F. nucleatum* levels were associated with a shorter survival in oesophageal cancer (Yamamura et al. 2016) and a poor response to neoadjuvant chemotherapy (Yamamura et al. 2019). Similar to oesophageal cancer, the presence of *F. nucleatum* in pancreatic tumour was found to be correlated with decreased survival rates (Alkharaan et al. 2020). In addition, *F. nucleatum* has the potential to enhance the invasiveness and metastatic capabilities of gastric cancer, consequently leading to a detrimental impact on the patient prognosis (Hsieh et al. 2021).

However, limited information is available regarding *Fusobacterium* species other than *F. nucleatum* and their potential contributions to human conditions. According to the List of Prokaryotic names with Standing in Nomenclature (LPSN), a total of 21 species are recognised in the *Fusobacterium* genus. In addition to *F. nucleatum* , several other species, including *F. necrophorum* (Riordan 2007), *F. gonidiaformans* (Citron 2002), *F. periodonticum* (Strauss et al. 2008), *F. mortiferum* , *F. ulcerans* (Adriaans and Shah 1988) and *F. varium* (Manson McGuire et al. 2014; Ohkusa et al. 2002, 2003), have been identified in human samples. Chan et al. demonstrated that non‐nucleatum *Fusobacteria* are the predominant taxa in the Southern Chinese population with and without cancer, and several of these taxa, especially *F. varium* and *F. ulcerans* , were enriched in CRC patients (Yeoh et al. 2020). Geva‐Zatorsky et al. (2017) reported that *F. varium* demonstrates a more pronounced phenotypic impact on host compared to *F. nucleatum* . Our previous study also indicated that non‐nucleatum types of *Fusobacterium* are dominant in the Southern Chinese population with distinctive correlations to host diseases (He et al. 2021). The findings of these studies suggest that the differences in detection method may impact the identification of microbial targets for diagnosis and treatment within specific populations. This underscores the urgent need to develop a high‐precision detection approaches for the identification of common *Fusobacterium* species and subspecies. Recent researches conducted by Bi et al. (2021) and Krieger et al. (2024) have resulted in the development of PCR‐based detection methods specifically targeting four subspecies of *F. nucleatum* (Table S1).

In the study, by taking advantage of large‐scale comparative genomics, we identified species and subspecies‐specific genetic markers in *Fusobacterium*, and subsequently developed a high‐precision multiplex PCR assay that allows easy differentiation of five species of *Fusobacterium* ( *F. nucleatum* , *F. mortiferum* , *F. ulcerans* , *F. varium* and *F. necrophorum* ) and four subspecies of *F. nucleatum* (*nucleatum*, *animalis*, *vincentii* and *polymorphum*). By employing the multiplex PCR method, we investigated the prevalent features of *Fusobacterium* communities in the Southern Chinese population as well as the tumours and faeces of cancer patients.

## 2 Materials and Methods

### 2.1 Bacterial Strains

*F. nucleatum* subsp. *nucleatum* (ATCC 25586), *F. nucleatum* subsp. *nucleatum* (ATCC 23726), *F. nucleatum* subsp. *polymorphum* (ATCC 10953), *F. nucleatum* subsp. *vincentii* (ATCC 49256), *F. nucleatum* subsp. *animals* (ATCC 51191), *F. necrophorum* ATCC (25286), *Lactobacillus iners* (ATCC 55195), *Bacteroides thetaiotaomicron* (ATCC 29148) were from American Type Culture Collection (ATCC, USA). *Escherichia coli* CMCC (B) 44102 was from National Center for Medical Culture Collections (CMCC, China). *Bacteroides acidifaciens* (DSM 15896) was from German Collection of Microorganisms and Cell Cultures (DSMZ, Germany). *Bacteroides fragilis* (BNCC 336948) was from BeNa Culture Collection (BNCC, China). *F. mortiferum* , *F. varium* , *F. ulcerans, Bifidobacterium longum*, *Lactobacillus paracasei* , *Enterococcus faecalis* , *Enterococcus avium* , *Staphylococcus epidermidis* , *Lactobacillus salivarius* , *Streptococcus salivarius* , *Bacteroides ovatus* , *Bifidobacterium pseudocatenulatum* , *Pediococcus pentosaceus* , *Klebsiella pneumoniae* , *Enterococcus faecium* , *Citrobacter freundii* , *Bacteroides plebeius* , *Slackia piriformis* , *Streptococcus agalactiae* , *Lactobacillus mucosae* , *Parabacteroides timonensis*, *Enterococcus gallinarum* and *Bacillus cereus* were isolated from clinical faecal samples, and confirmed by microbial mass spectrometry detection system (QuanTOF I, China) and 16S rRNA gene sequencing. *Fusobacterium*, *Escherichia coli* and *Bacteroides fragilis* were cultivated on Columbia agar supplemented with 5% sheep blood (Dijing, Guangzhou, China). *Lactobacillus iners* was cultivated on TSA or TSB medium supplemented with 5% sheep blood. *Bacteroides acidifaciens* was cultivated on GAM medium. *Bacteroides thetaiotaomicron* was cultivated on TSA medium supplemented with 5% sheep blood. TSA, TSB and GAM medium were all ordered from Guangdong Huankai Microbial Science and Technology Co. Ltd. (Guangzhou, China). *Escherichia coli* and *Lactobacillus iners* were cultured aerobically at 37°C. Other strains were cultivated at 37°C in a multi‐purpose incubator (GeneScience, Chongqing, China).

### 2.2 *Fusobacterium* Genomic Analysis and Species‐Specific Genetic Markers Identification

A total of 217 Fusobacterium genomes, including 6 *F. nucleatum* subsp. nucleatum, 20 *F. nucleatum* subsp. polymorphum, 10 *F. nucleatum* subsp. vincentii, 17 *F. nucleatum* subsp. animals, 38 *F. mortiferum* , 16 *F. ulcerans* , 25 *F. varium* and 85 *F. necrophorum* , were retrieved from the NCBI (National Center of Biotechnology Information) database. Duplicated genomes, unannotated genomes or those not meeting the RefSeq criteria were excluded. Finally, 214 unique genomes were included for the analysis (Table S2).

*Fusobacterium* genomes were subjected to IPGA (Integrated Prokaryotes Genome and pan‐genome Analysis) for pangenome analysis (Liu et al. 2022). The downstream comparative genomic analysis included phylogenetic analysis, core gene allele analysis and genome annotation analysis. The sequence regions, which were highly conserved within the species and largely differed in different species simultaneously, were selected as alternatives of core genes. The core genes sequences were integrated and clustered at 100% similarity using cd‐hit‐est. Kraken analysis identified specific sequences, which were then annotated using Prokk's annotation file in IPGA. The selected core genes were compared against the NCBI nucleotide database to exclude those with homologous sequences to other organisms. The core gene sequences ultimately included were defined as species or subspecies‐specific markers.

### 2.3 The Design of PCR Primers and Probes

Based on above identification of species‐specific sequences after *Fusobacterium* genomic analysis, the PCR primers and probes were designed for different *Fusobacterium* species and subspecies. All primer and probes used in this study and their main characteristics are listed in Table 1. Here, primers were designed to have a similar melting temperature (*T*_m_). A putative non‐specific amplification was assessed by comparing primer sequences with the NCBI nucleotide database. The primers and probes were analysed for the formation of autodimers, crossover dimers, and hairpin structures using Primer Express. Resulting primers had an annealing temperature between 48.3°C and 64.0°C, a GC content of 21.0%–43.0%, and a length of 20 to 33 nucleotides. The probes revealed an annealing temperature between 64.8°C and 70.0°C, a GC content of 29.0%–50.0%, and a length between 20 and 26 nucleotides. All oligonucleotides and probes were synthetised by Guangzhou Heyuan Biotechnologies Co. Ltd.

**TABLE 1:** Primer and probe sequences used for real‐time PCR detection of *Fusobacterium* ^a^.

Target	Target gene	Primer name	Sequences (5′–3′)	Amplicon sizes (bp)	GC%	*T*_m_ (°C)
*F. mortiferum*	braC_1 (cluster_727) (GCA_003019315.1)	FM‐F	GAGTTCTACTATTAAAGTAGGAGGAA	96	35	50.4
		FM‐R	TCAAAGGCTAATTTAGCTCC		40	50.6
		FM‐P	Texas Red‐CCTTTAACTGGTTCTGCTGC‐MGB		50	70.0
*F. varium*	dhaD (cluster_332) (GCA_003019655.1)	FV‐F	ACATCTGCCTTATCTGTTGT	161	40	48.3
		FV‐R	TAAAATTTGGCAAGTGTATCTCC		35	54.1
		FV‐P	FAM‐ATTTTTTGATTTTCCTTCTCCTCCTA‐MGB		31	70.0
*F. ulcerans*	yeeO_2 (cluster_7004) (GCA_003019675.1)	FU‐F	ATAGATCTGACTTTATTGAAAAAAACTA	115	21	52.2
		FU‐R	TTCCCAAAGGATTTACTGCTC		43	54.8
		FU‐P	Texas Red‐TATAGCTTGTCTTATGCAGTTCA‐MGB		35	70.0
*F. necrophorum*	hadC_1 (cluster_1389) (GCA_003732525.1)	FNE‐F	TGGCCAAAGAGTATTTTCCACCATTC	130	42	64.0
		FNE‐R	AGGGAAATTAAGGAGTCCGTCAT		43	58.1
		FNE‐P	FAM‐TGTATGTTCCGTTCAATCCCAGTTCC‐MGB		46	64.8
Subsp. nucleatum	Fnn_1803 (cluster_1803) (GCA_003019295.1)	Fn‐F	CAAGCAACTGAAAATGCTTTAAAAG	216	32	57.6
		Fn‐R	TCCAGGTAAGGAAATTACACCTACTG		42	57.9
		Fn‐P	VIC‐ACATAGTAAATTGGCTGATT‐MGB		30	66.0
Subsp. animals	Fna_2959 (cluster_2959) (GCA_001296145.1)	Fa‐F	TGGAATGGAATATGAAACTATGGACTA	87	33	58.1
		Fa‐R	GAAGGATGAAATGCTGGACATCT		43	58.2
		Fa‐P	VIC‐TCAGATATGGGAAATGTAAG‐MGB		35	68.0
Subsp. vincentii	Fnv_2681 (cluster_2681) (GCA_002764055.1)	Fv‐F	GAGGCTATTGCAAATTAAACTGTTAAA	123	30	57.5
		Fv‐R	CTTTACCACTATTATAAACTAAATAAATGAGAC		24	54.5
		Fv‐P	Texas Red‐TAGCTAGTACTGAACAATTT‐MGB		30	69.0
Subsp. polymorphum	Fnp_2834 (cluster_2834) (GCA_001457555.1)	Fp‐F	TCTACTGTAATAGTTACAAACTCTGCACC	134	38	56.8
		Fp‐R	TTAGGAAATCTTTTAGAAGCAAAAACA		26	57.2
		Fp‐P	FAM‐AGTTTCTTCTTTAAGTAGTCCAAA‐MGB		29	68.0
GAPDH^a^		GAPDH‐F	GGGAAACTGTGGCGTGAT	158	56	55.4
		GAPDH‐R	AGGTCCACCACTGACACGT		58	54.7
		GAPDH‐P	CY5‐CGGGCCTCTCCAGATCATC‐BHQ2		63	59.1

^a^ *Fusaobacterium* includes four species of *Fusobacterium* ( *F. mortiferum* , *F. ulcerans* , *F. varium* and *F. necrophorum* ) and four subspecies of *F. nucleatum* (*nucleatum*, *animalis*, *vincentii* and *polymorphum*); GAPDH was selected as the internal standard.

### 2.4 The Development of Multiplex Real‐Time PCR Assay

PCR was performed with the real‐time PCR detection system ABI 7500 (Applied Biosystems; Thermo Fisher Scientific Inc., USA) using the following reaction conditions: UDG enzyme activation step at 50°C for 2 min and initial denaturation step at 95°C for 5 min and 45 cycles of amplification (denaturation at 95°C for 5 s and annealing/extension at 60°C for 35 s). Single‐target fluorescent PCR was employed to screen optimal primer‐probe combinations for each target. These were then randomly combined into multiplex systems for further screening, identifying the best configurations of different targets. The final steps involved determining primer and probe concentration gradients (100–1000 nM), optimising the quantities of primers and probes in multiplex systems, and selecting combinations with both favourable amplification profiles and no cross‐reactions to establish effective multiplex reaction systems. Here, the primers of *Fusobacterium* species and subspecies, including *F. nucleatum* subsp. *nucleatum*, *F. nucleatum* subsp. *polymorphum, F. nucleatum* subsp. *vincentii*, *F. nucleatum* subsp. *animals*, *F. mortiferum* , *F. ulcerans* , *F. varium* and *F. necrophorum* , were assigned into different primer sets for verification, and three primer sets were eventually screened in the multiplex PCR detection system. Then, the multiplex real‐time PCR assays were evaluated for amplification efficiency, sensitivity and specificity. For the determination of reaction efficiencies, regression lines were created by plotting the quantification cycle (Cq) values versus the log of the target DNA concentration used for qPCR. The amplification efficiency (*E*) was calculated for each probe from the slopes using the formula:

$$E=\left(10^{–1/\text{slope}}-1\right)\times 100\%.$$

In order to evaluate the sensitivity of the qPCR assay, a DNA sample with 1 × 10^3^ copies/mL is tested in 20 wells, aiming for a 95% detection rate as the benchmark. *Escherichia coli, Lactobacillus iners, Bacteroides thetaiotaomicron, Bacteroides acidifaciens, Bacteroides fragilis*, along with non‐reactive tube controls, were utilised as samples to evaluate the specificity of qPCR reagents. The relative abundance of these targets was calculated in reference to total bacterial DNA using the following primers: P891F (5′‐TGGAGCATGTGGTTTAATTCGA‐3′), P1033R (5′‐TGCGGGACTTAACCCAACA‐3′) and UniProbe (5′‐FAM‐CACGAGCTGACGACARCCATGCA‐BHQ‐3′) (Yang et al. 2002).

### 2.5 DNA Preparation

Bacterial DNA was prepared with bacteria DNA extraction kit (HiPure Bacterial DNA Kit, China) according to manufacturer's instructions. In the cohorts, DNA was extracted from homogenised fractions of faeces and tissues using the QIAamp PowerFecal Pro DNA Kit (QIAGEN GmbH, Hilden, Germany) and MagPure Universal DNA Precast Kit (MAGEN, China) according to manufacturer's instructions. The concentration of extracted DNA was determined using a spectrophotometer (Tiangen, Beijing, China).

### 2.6 Study Design and Study Population

In this study, we included three cohorts to elucidate the prevalence and clinical relevance of *Fusobacterium* in both normal population and cancer patients. This study was approved by the Ethics Committee of Zhujiang Hospital, Southern Medical University (2023‐KY‐173‐01) and the Chinese Center for Disease Control and Prevention (201519‐A).

In cohort A (Figure S1A), a total of 498 subjects were randomly sampled from GGMP (Guangdong Gut Microbiome Project), and were included in the present analysis as part of our cohort study to profile the prevalence of *Fusobacterium* in Southern Chinese population. We previously described the population included in the GGMP (He et al. 2021; He, Wu, Zheng, et al. 2018; He, Wu, Wu, et al. 2018).

In cohort B (Figure S1B), a total of 397 participants, including 308 cases with solid cancer and 96 cases without cancer, were enrolled at Zhujiang Hospital, Southern Medical University in Guangdong, China from Mar 01, 2023 to Sep 31, 2023. Participants with solid tumour (cases) were enrolled within 72 h of admission, and no antibiotics or probiotics were allowed within 2 weeks before or during the study period. Participants without solid tumour (controls) were recruited from the physical examination centre. This clinical cohort was used for the analysis of compositional features of *Fusobacterium* communities in gut of solid tumour patients.

In cohort C (Figure S1C), CRC and lung cancer patients receiving surgical resection (> 18 years old, either sex) were included. Patients who had received antibiotics or probiotics within 2 weeks prior to sample collection were excluded. Ultimately, 21 of CRC participants with 46 samples were evaluated, despite 6 lacking tumour tissues, 6 lacking adjacent tissues and 5 lacking normal tissues. Meanwhile, 49 of lung cancer participants with 186 samples were analysed, despite 3 lacking tumour tissues, 3 lacking adjacent tissues, 4 lacking normal tissues and 1 lacking faeces. The matched tumour, adjacent tissues, normal tissues, and faeces were retrieved from the Biobank of Zhujiang Hospital to analyse the compositional features of *Fusobacterium* communities in these clinical samples. Moreover, the correlations between *Fusobacterium* and CRC and lung cancer were investigated.

### 2.7 Statistical Analysis

The results were analysed with GraphPad Prism (version 10), SPSS (version 19) and R (version 4.0.2). Chi‐square tests were used to compare rates, and Mann–Whitney U tests were used to compare measured values. Non‐parametric tests were used if data exhibited a non‐normal distribution. For comparisons among multiple groups, the Kruskal–Wallis H test followed by Dunn's test was used. Heatmaps were generated with the ChiPlot (https://www.chiplot.online/index.html). *p* < 0.05 was considered statistically significant.

## 3 Results

### 3.1 Large‐Scale Comparative Genomic Analysis Identified Species‐Specific Genetic Markers in *Fusobacterium*

**FIGURE 1:**
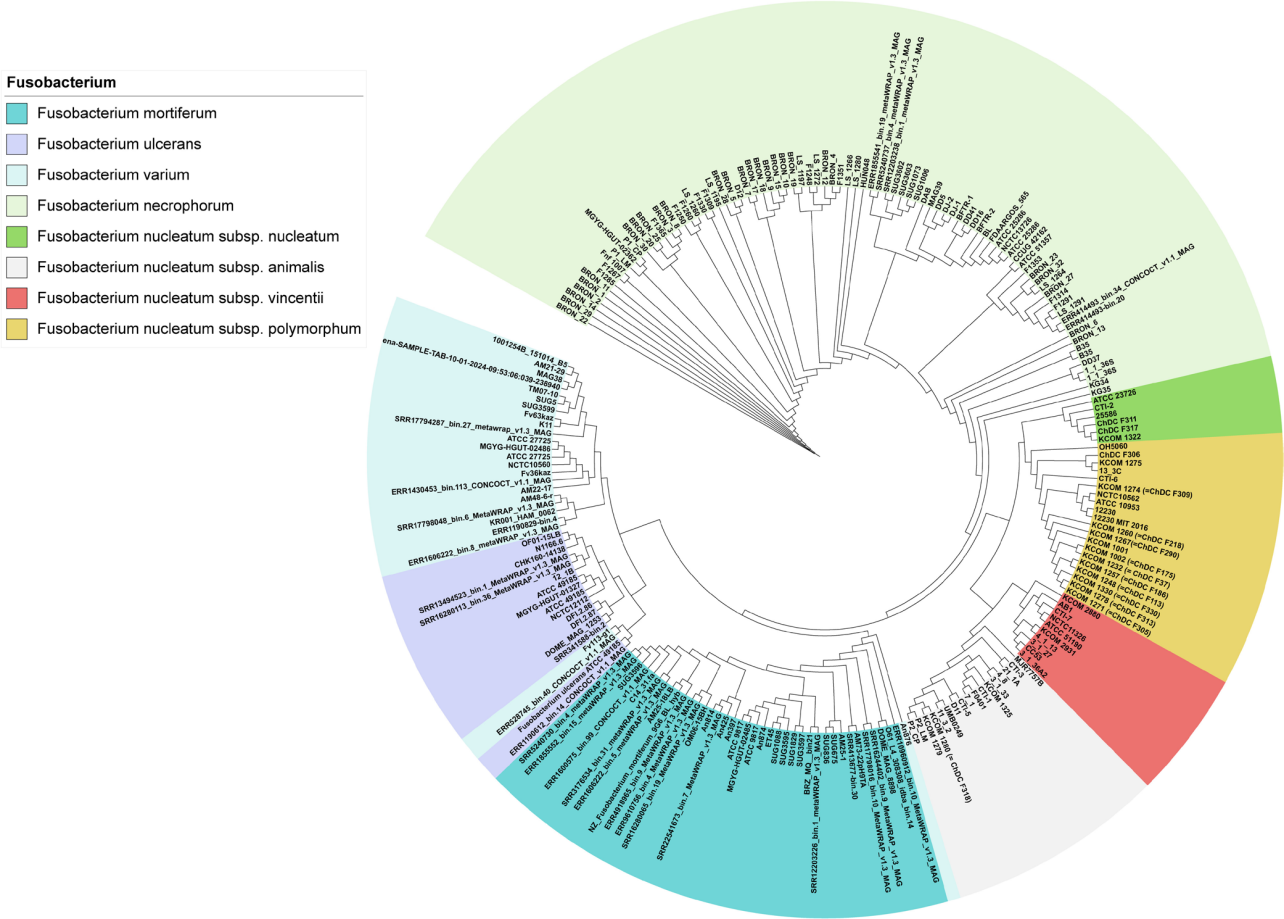
The phylogenetic tree of *Fusobacterium* species/subspecies. A total of 217 *Fusobacterium* genomes were analysed. The phylogenetic tree was generated by using IPGA with the maximum likelihood algorithm. Three strains formerly known as *F. varium* were reassigned as *F. ulcerans* and *F. mortiferum* , and excluded for subsequent investigation.

To develop a high‐precision multiplex PCR method that can concurrently identify five *Fusobacterium* species and four *F. nucleatum* subspecies in the microbiota, we sought to search for candidate taxonomic markers in *Fusobacterium* genomes. A total of 217 *Fusobacterium* genomes from NCBI database were employed (Table S2), and whole‐genome phylogenetic analysis was performed to ensure correct species and subspecies assignment. These strains of *Fusobacterium* formed eight clades in the phylogenetic tree, corresponding well to *F. nucleatum* subsp. *nucleatum*, *F. nucleatum* subsp. *polymorphum, F. nucleatum* subsp. *vincentii*, *F. nucleatum* subsp. *animals*, *F. mortiferum* , *F. ulcerans* , *F. varium* and *F. necrophorum* (Figure 1), but three strains, formerly known as *F. varium* , were reassigned as *F. ulcerans* Fv113‐g1, *ERR528745*_bin.40_CONCOCT_v1.1_MAG and *F. mortiferum* An876.

**FIGURE 2:**
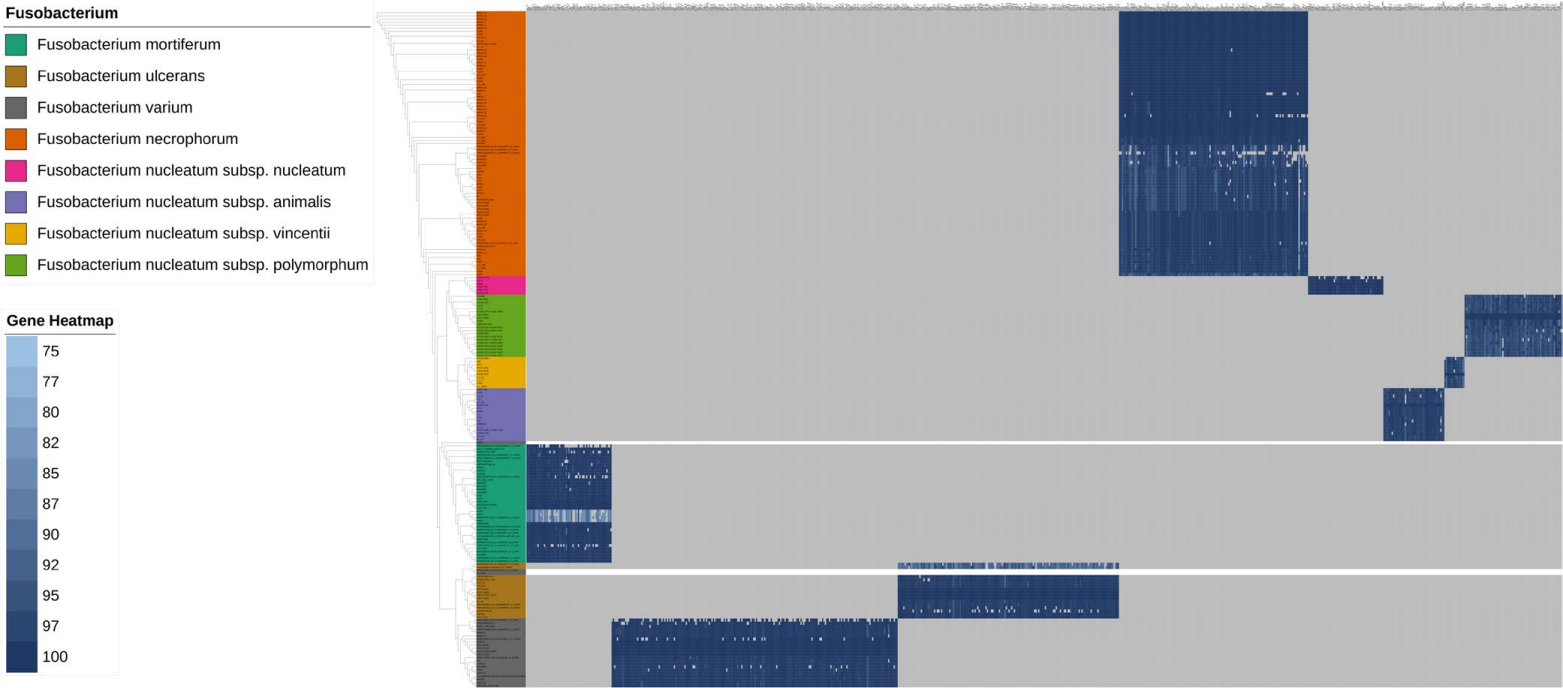
The identification of species‐specific genetic markers in *Fusobacterium*. The heatmap shows the occurrence and nucleotide‐level conservation of the identified species or subspecies‐specific markers. A total of 214 *Fusobacterium* genomes were used for the analysis of specific genetic markers. Strain names are listed to the left of the heatmap. Genes annotated with Prokka are listed on the heatmap. A colour gradient was employed to visualise the nucleotide identity between the reference sequences and their counterparts. ND, not detected.

A total of 214 *Fusobacterium* genomes, including *F. nucleatum* subsp. *nucleatum* (6 strains), *F. nucleatum* subsp. *polymorphum* (20 strains), *F. nucleatum* subsp. *vincentii* (10 strains), *F. nucleatum* subsp. *animals* (17 strains), *F. mortiferum* (38 strains), *F. ulcerans* (16 strains), *F. varium* (22 strains) and *F. necrophorum* (85 strains), were analysed by IPGA to identify core genes that can act as species or subspecies‐specific genetic markers. Specific genes or non‐coding sequences in all genomes of one species/subspecies of *Fusobacterium* but absent in other species/subspecies of *Fusobacterium* were selected as core genes or sequences. As a result, 56, 72, 15, 45, 63, 164, 212 and 140 genes were found to be specific to *F. nucleatum* subsp. *nucleatum*, *F. nucleatum* subsp. *polymorphum*, *F. nucleatum* subsp. *vincentii*, *F. nucleatum* subsp. *animals*, *F. mortiferum* , *F. ulcerans* , *F. varium* and *F. necrophorum* (Figure 2). In regard with specific fragments of *F. nucleatum* subsp. *vincentii*, Fnv_2681 was found located in the non‐coding region in addition to the prokka annotated core genes. It was consistent with the primer fragment designed by Bi et al. (2021). Therefore, we incorporated this core fragment into the methodology. Importantly, inter‐ and intra‐subspecies comparisons showed that the identified markers exhibited strict species/subspecies specificity, with highly conserved sequences (with 77.4%–100% identities to reference strains; median: 99.7%) (Figure 2). Homologues of these markers were not found in other organisms, as examined in the NCBI nucleotide database.

### 3.2 Development of High‐Precision Multiplex PCR Assay for the Identification of *Fusobacterium* Species and Subspecies

Based on above screening of specific core sequences in *Fusobacterium* genomes, specific primers and probes were designed for different *Fusobacterium* species and subspecies (Table 1). To select highly specific primers for *F. nucleatum* subsp. *nucleatum* and subsp. *vincentii*, we employed the IPGA platform to conduct a comparative genomic analysis. This analysis confirmed that the genetic markers reported by Bi et al. (2021) were distinct and applicable within our strain sets. As the primers from Bi et al. (2021) were designed against these confirmed markers and have been extensively validated, we applied them in our multiplex PCR system, where they performed extremely well (Table 1). For practical use in multiplex PCR system, all primers have similar Tms, and the amplicon sizes are 80 bp–200 bp (mostly < 100 bp). To achieve best sensitivity and specificity, different concentrations of primers and probes were tested to determine the optimal conditions. The best concentrations of primers and probes (100–1000 nM) were determined for the multiplex PCR system (Table 2). Then, the primer sets were screened in the multiplex PCR detection system, and three primer sets were eventually selected, including Primer Set A ( *F. nucleatum* subsp. *polymorphum*, *F. nucleatum* subsp. *nucleatum* and *F. nucleatum* subsp. v*incentii*), Primer Set B ( *F. necrophorum* , *F. nucleatum* subsp. *animals* and *F. mortiferum* ) and Primer Set C ( *F. ulcerans* and *F. varium* ) (Table 2). Moreover, all primer sets showed excellent specificity (Table 3).

**TABLE 2:** The best concentrations of primers and probes determined for the multiplex PCR system.

Primer sets	Target	Primer name	Concentrations (nM)
Primer set A	Subsp. polymorphum	Fp‐F	200
		Fp‐R	200
		Fp‐P	267
	Subsp. nucleatum	Fn‐F	200
		Fn‐R	200
		Fn‐P	200
	Subsp. vincentii	Fv‐F	267
		Fv‐R	267
		Fv‐P	333
Primer set B	*F. necrophorum*	FNE‐F	200
		FNE‐R	200
		FNE‐P	267
	Subsp. *animals*	Fa‐F	333
		Fa‐R	333
		Fa‐P	133
	*F. mortiferum*	FM‐F	200
		FM‐R	200
		FM‐P	267
Primer set C	*F. varium*	FV‐F	267
		FV‐R	267
		FV‐P	200
	*F. ulcerans*	FU‐F	267
		FU‐R	267
		FU‐P	267

**TABLE 3:** The screening result of PCR primers specificity.

No.	Name of the strain	Source code no.#	Result of PCR test		
			Primer set A^a^	Primer set B^a^	Primer set C^a^
1	*F. ulcerans*	Isolates	−	−	+
2	*F. varium*	Isolates	−	−	+
3	*F. mortiferum*	Isolates	−	+	−
4	*F. necrophorum*	ATCC 25286	−	+	−
5	Subsp. polymorphum	ATCC 10953	+	−	−
6	Subsp. nucleatum	ATCC 25586	+	−	−
7	Subsp. vincentii	ATCC 49256	+	−	−
8	Subsp. animals	ATCC 51191	−	+	−
9	*Escherichia coli*	CMCC (B) 44,102	−	−	−
10	*Lactobacillus iners*	ATCC 55195	−	−	−
11	*Bacteroides thetaiotaomicron*	ATCC 29148	−	−	−
12	*Bacteroides acidifaciens*	DSM 15896	−	−	−
13	*Bacteroides fragilis*	BNCC336948	−	−	−
14	*Bifidobacterium longum*	Isolates	−	−	−
15	*Lactobacillus paracasei*	Isolates	−	−	−
16	*Enterococcus faecalis*	Isolates	−	−	−
17	*Enterococcus avium*	Isolates	−	−	−
18	*Staphylococcus epidermidis*	Isolates	−	−	−
19	*Lactobacillus salivarius*	Isolates	−	−	−
20	*Streptococcus salivarius*	Isolates	−	−	−
21	*Bacteroides ovatus*	Isolates	−	−	−
22	*Bifidobacterium pseudocatenulatum*	Isolates	−	−	−
23	*Pediococcus pentosaceus*	Isolates	−	−	−
24	*Klebsiella pneumoniae*	Isolates	−	−	−
25	*Enterococcus faecium*	Isolates	−	−	−
26	*Citrobacter freundii*	Isolates	−	−	−
27	*Bacteroides plebeius*	Isolates	−	−	−
28	*Slackia piriformis*	Isolates	−	−	−
29	*Streptococcus agalactiae*	Isolates	−	−	−
30	*Lactobacillus mucosae*	Isolates	−	−	−
31	Parabacteroides timonensis	Isolates	−	−	−
32	*Enterococcus gallinarum*	Isolates	−	−	−
33	*Bacillus cereus*	Isolates	−	−	−

^a^ The information of primer sets was shown in Table 2.

To assess the amplification efficiency, we employed a combination of qPCR and ddPCR to determine the correlation between Cq values and DNA concentrations. Here, 1 × 10^7^ copies/mL of DNA mixture were diluted in 10‐fold gradient to generate five concentration gradients ranging from 1 × 10^3^ to 10^7^ copies/mL. Bacterial DNA within the range from 1 × 10^4^ to 10^7^ copies/mL were identified in PCR reaction tubes A–C, with amplification efficiencies ranging from 90.465% to 101.183% and *R* ^2^ values ranging from 0.995 to 1 (Table 4). In order to evaluate the sensitivity of qPCR assay, 1 × 10^3^ copies/mL of target DNA mixture were used, and 20 replicates were tested, and 95% of detection rate was used as the critical value. When 1 × 10^3^ copies/mL of target DNA were detected, the Cq values of each channel of each reaction tube ranged from 34.84 to 41.46, with missed detection of *F. nucleatum* subsp. *vincentii* and *F. mortiferum* , and all target DNA of PCR reaction tube 1–3 met the detection rate of 95% (Table 5). Hence, the cut‐off value of Cq range was established as 42, with a sensitivity of 1 × 10^3^ copies/mL of DNA for the reaction system. In addition, 49 of human faecal samples were employed to assess the sensitivity and specificity of the multiplex PCR assay in complex backgrounds compared to amplicon sequencing (Tables S3–, S11).

**TABLE 4:** The correlation between Cq values and DNA concentration.

Probe and primer	Template DNA from	Concentration of target DNA (copies/mL)	Mean Cq value	Efficiency/*R* ^2^ value
Primer set A	Subsp. polymorphum	1 × 10^7^	23.46 ± 0.34	90.465
		1 × 10^6^	26.68 ± 0.09	0.995
		1 × 10^5^	30.09 ± 0.21	
		1 × 10^4^	33.95 ± 0.16	
	Subsp. nucleatum	1 × 10^7^	22.69 ± 0.19	93.099
		1 × 10^6^	26.01 ± 0.08	0.998
		1 × 10^5^	29.40 ± 0.15	
		1 × 10^4^	33 ± 0.14	
	Subsp. vincentii	1 × 10^7^	20.8 ± 0.2	97.220
		1 × 10^6^	23.98 ± 0.12	0.998
		1 × 10^5^	27.24 ± 0.12	
		1 × 10^4^	30.86 ± 0.11	
Primer set B	*F. necrophorum*	1 × 10^7^	22.78 ± 0.11	101.183
		1 × 10^6^	26.11 ± 0.09	1
		1 × 10^5^	29.49 ± 0.16	
		1 × 10^4^	32.69 ± 0.05	
	Subsp. *animals*	1 × 10^7^	21.75 ± 0.4	96.323
		1 × 10^6^	25.16 ± 0.29	0.999
		1 × 10^5^	28.58 ± 0.52	
		1 × 10^4^	31.97 ± 0.35	
	*F. mortiferum*	1 × 10^7^	25.07 ± 0.15	100.164
		1 × 10^6^	28.36 ± 0.22	0.999
		1 × 10^5^	31.9 ± 0.15	
		1 × 10^4^	35.31 ± 0.53	
Primer set C	*F. varium*	1 × 10^7^	22.66 ± 0.22	94.294%
		1 × 10^6^	26.26 ± 0.09	0.999
		1 × 10^5^	29.29 ± 0.12	
		1 × 10^4^	33.10 ± 0.19	
	*F. ulcerans*	1 × 10^7^	23.63 ± 0.01	98.391%
		1 × 10^6^	27.28 ± 0.002	0.998
		1 × 10^5^	30.41 ± 0.1	
		1 × 10^4^	33.79 ± 0.25	

**TABLE 5:** The sensitivity of multiplex PCR assays.

Probe and primer	Template DNA from	Concentration of target DNA (copies/mL)	Cq value
Primer set A	Subsp. polymorphum	1 × 10^3^	36.83–39.47
	Subsp. nucleatum	1 × 10^3^	35.84–37.79
	Subsp. vincentii	1 × 10^3^	37.23–41.46
Primer set B	*F. necrophorum*	1 × 10^3^	36.47–38.77
	Subsp. *animals*	1 × 10^3^	35.99–38.02
	*F. mortiferum*	1 × 10^3^	36.41–40.24
Primer set C	*F. varium*	1 × 10^3^	34.84–37.16
	*F. ulcerans*	1 × 10^3^	36.00–38.57

### 3.3 Application of the Multiplex PCR Assay for Profiling *Fusobacterium* in Southern Chinese Population

**FIGURE 3:**
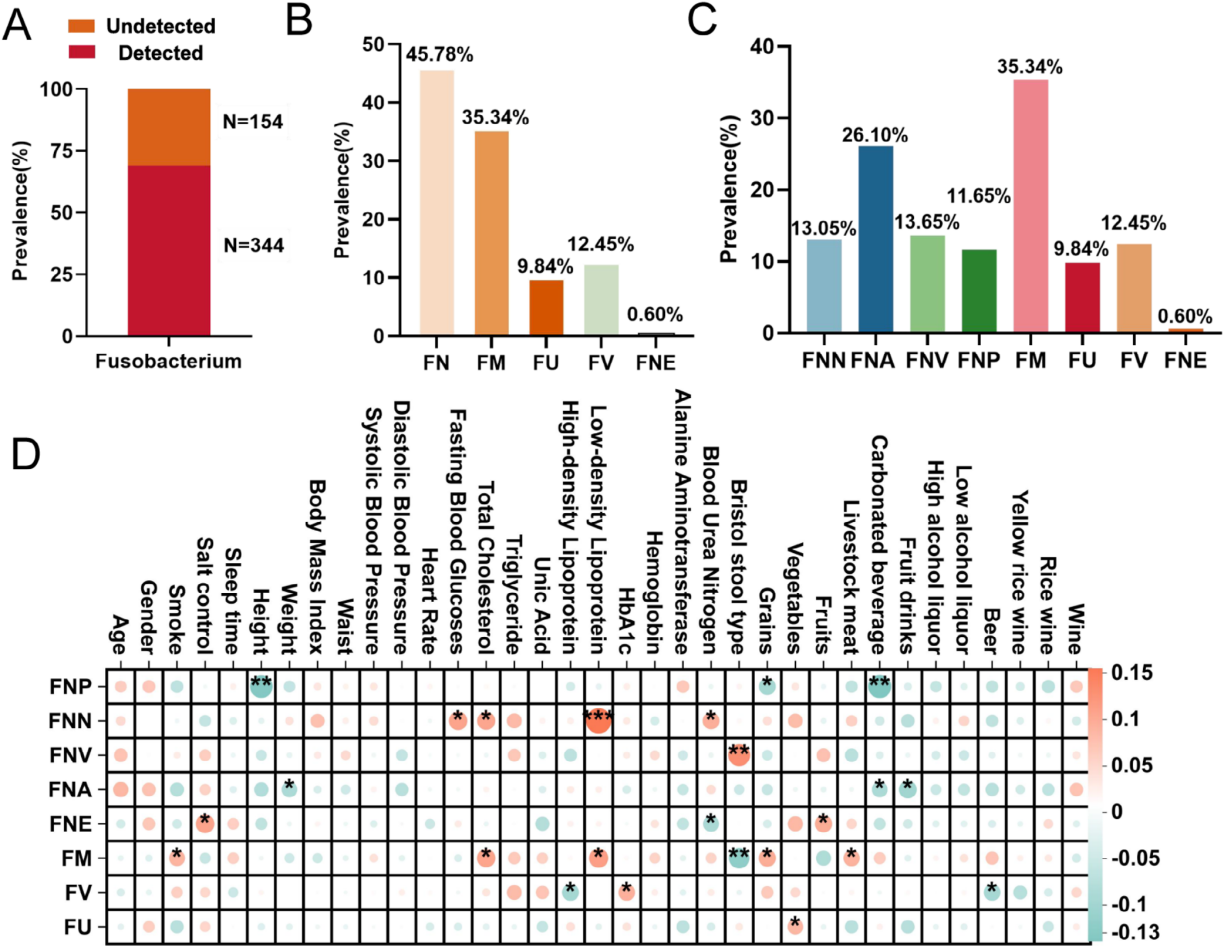
The prevalence of *Fusobacterium* in Southern Chinese population with correlations to host conditions. (A) The prevalence of *Fusobacterium* in Southern Chinese population; (B) The prevalence of *Fusobacterium* species and (C) subspecies in Southern Chinese population; (D) The correlations between *Fusobacterium* and host parameters. The correlation analysis is spearman analysis. A higher absolute value of the correlation coefficient signifies a stronger correlation, with red denoting a positive correlation and blue indicating a negative correlation. The size of the nodes and the depth of their colour represent the strength of the correlation, where larger nodes and darker hues correspond to stronger correlations. An asterisk (*) denotes the *p*‐value. FM, *Fusobacterium mortiferum* ; FN, *Fusobacterium nucleatum* ; FNA, *Fusobacterium nucleatum* subsp. *animalis*; FNE, *Fusobacterium necrophorum* .; FNN, *Fusobacterium nucleatum* subsp. *nucleatum*; FNP, *Fusobacterium nucleatum* subsp. *polymorphum*; FNV, *Fusobacterium nucleatum* subsp. *vincentii*; FU, *Fusobacterium ulcerans* ; FV, *Fusobacterium varium* .

The new‐developed multiplex PCR assay was conducted to investigate the prevalence of *Fusobacterium* in Southern Chinese population and its correlations to host conditions. Here, 498 individuals were randomly sampled from GGMP (He et al. 2021; He, Wu, Zheng, et al. 2018; He, Wu, Wu, et al. 2018), which were included in the present study representative of the Southern Chinese population (Figure S1A). We found that *Fusobacterium* was present in 69.08% of the study population (Figure 3A). Intriguingly, *F. nucleatum* was most prevalent (45.78%) in Southern Chinese population, followed by *F. mortiferum* (35.34%), *F. varium* (12.45%), *F. ulcerans* (9.84%) and *F. necrophorum* (0.60%) (Figure 3B). At the subspecies level of *F. nucleatum* subsp. *animals* was most prevalent (26.10%), followed by subsp. *vincentii* (13.65%), subsp. *nucleatum* (13.05%) and subsp. *polymorphum* (11.65%) (Figure 3C).

To explore the correlations of *Fusobacterium* to host conditions, correlation analyses were performed. We discovered significant correlations between both *F. nucleatum* subsp. *nucleatum* and *F. mortiferum* and both low‐density lipoprotein cholesterol (LDL) and total cholesterol, and also *F. nucleatum* subsp. *nucleatum* and fasting blood glucose and blood urea nitrogen (Figure 3D). *F. nucleatum* subsp. *vincentii* was linked to bristol stool type, *F. necrophorum* to salt control and fruits, *F. mortiferum* to smoke, grains and livestock meat, *F. ulcerans* to vegetables and *F. varium* to HbA1c (glycosylated haemoglobin, type A1C) (Figure 3D). Conversely, *F. nucleatum* subsp. *polymorphum* showed a negative correlation with height, grains and carbonated beverage, *F. nucleatum* subsp. animals with weight, carbonated beverage and fruit drinks, *F. necrophorum* with blood urea nitrogen, *F. mortiferum* with bristol stool type and *F. varium* with high‐density lipoprotein cholesterol and beer (Figure 3D).

### 3.4 Application of the Multiplex PCR Assay for Profiling *Fusobacterium* and Its Diagnostic Performance in Cancer Patients

Next, we studied the prevalent features of *Fusobacterium* communities in faecal samples of clinical cancer cohort (Figure S1B). Table S12 showed the characteristics of study participants in cohort B, which were compared between case group and control group. The case group had older age (60.50 (53.00–69.00), *p* < 0.001), lower BMI (body mass index) (23.03 (20.06–24.91), *p* < 0.001) and higher HR (heart rate) (82.50 (75.00–92.00), *p* = 0.005). As for the laboratory results, the case group had lower UA (uric acid) (333.00 (268.00–401.25), *p* < 0.001), lower Hb (haemoglobin) (120.00 (109.75–134.00), *p* < 0.001), lower ALT (alanine aminotransferase) (17.00 (12.00–28.00), *p* < 0.001), higher positive rate of FOB (faeces occult blood) (62 (24.41%), *p* < 0.001) and higher CEA (carcinoembryonic antigen) (2.60 (1.60–4.75), *p* < 0.001).

**FIGURE 4:**
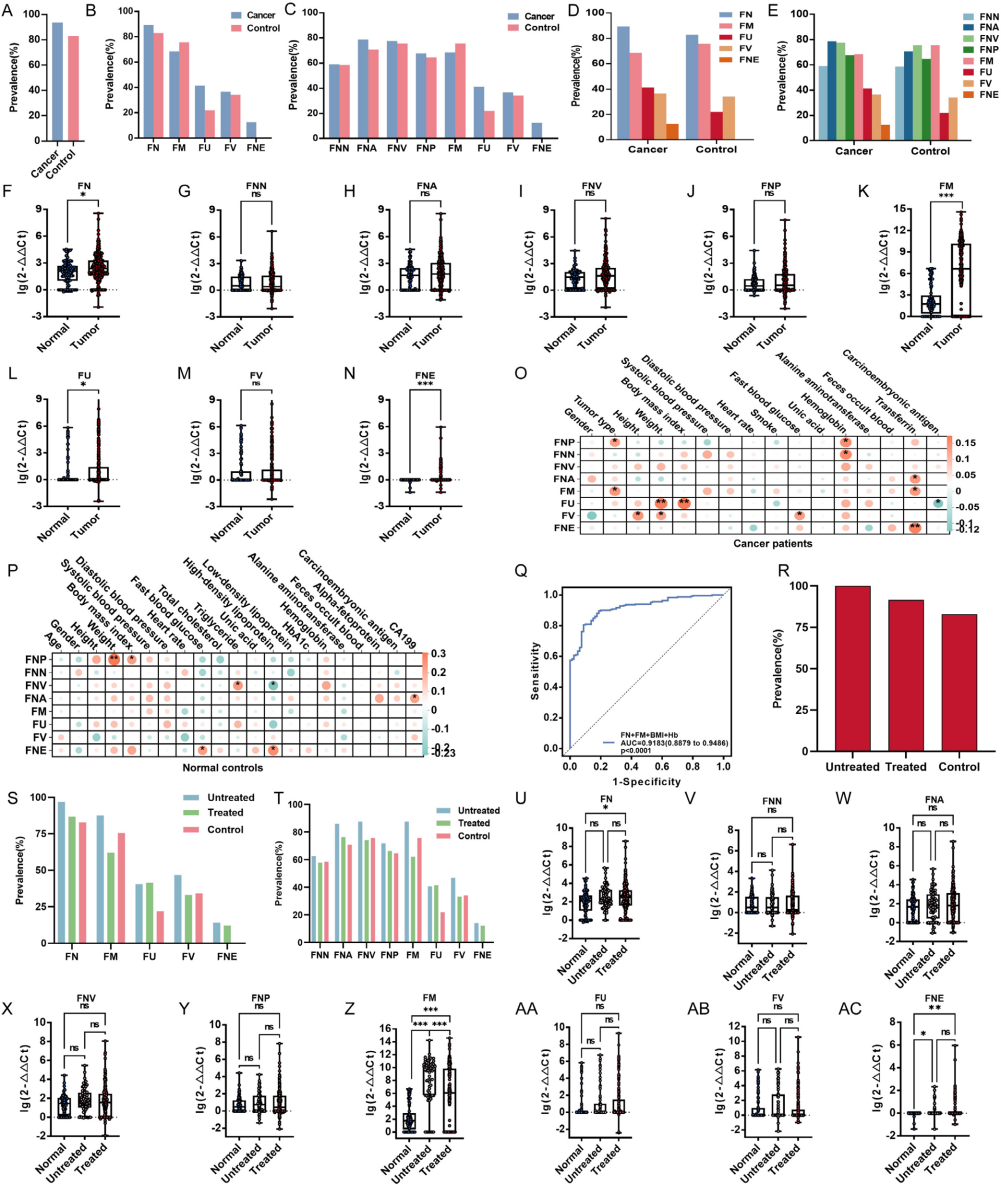
The prevalence of *Fusobacterium* in faeces of cancer patients with correlations to host conditions. (A) The prevalence of *Fusobacterium* in cancer patients (*n* = 254) and healthy controls (*n* = 82); (B–E) The prevalence of *Fusobacterium* species and subspecies in cancer patients (*n* = 254) and healthy controls (*n* = 82); (F–N) The relative abundance of FN (F), FNN (G), FNA (H), FNV (I), FNP (J), FM (K), FU (L), FV (M) and FNE (N) in faecal samples of cancer patients (*n* = 254) and healthy controls (*n* = 82). Mann–Whitney test. (O) The correlations between *Fusobacterium* and host parameters in cancer patients (*n* = 254). The correlation analysis is spearman analysis. A higher absolute value of the correlation coefficient signifies a stronger correlation, with red denoting a positive correlation and blue indicating a negative correlation. The size of the nodes and the depth of their colour represent the strength of the correlation, where larger nodes and darker hues correspond to stronger correlations. An asterisk (*) denotes the *p*‐value. (P) The correlations between *Fusobacterium* and host parameters in healthy controls (*n* = 82). The correlation analysis is spearman analysis. A higher absolute value of the correlation coefficient signifies a stronger correlation, with red denoting a positive correlation and blue indicating a negative correlation. The size of the nodes and the depth of their colour represent the strength of the correlation, where larger nodes and darker hues correspond to stronger correlations. An asterisk (*) denotes the *p*‐value. (Q) The receiver operating characteristic (ROC) curve for predicting cancers. AUC, the area under the ROC curve. The 95% confidence intervals are shown in brackets. (R) The prevalence of *Fusobacterium* in untreated (*n* = 64), treated (190) and control (*n* = 82) group. (S, T) The prevalence of *Fusobacterium* species and subspecies in untreated (*n* = 64), treated (190) and control (*n* = 82) group. (U–AC) The relative abundance of FN (U), FNN (V), FNA (W), FNV (X), FNP (Y), FM (Z), FU (AA), FV (AB) and FNE (AC) in faecal samples of untreated (*n* = 64) and treated (*n* = 190) cancer patients and control group (*n* = 82). Kruskal–Wallis H test followed by Dunn's test. FM, *Fusobacterium mortiferum* ; FN, *Fusobacterium nucleatum* ; FNA, *Fusobacterium nucleatum* subsp. *animalis*; FNE, *Fusobacterium necrophorum* .; FNN, *Fusobacterium nucleatum* subsp. *nucleatum*; FNP, *Fusobacterium nucleatum* subsp. *polymorphum*; FNV, *Fusobacterium nucleatum* subsp. *vincentii*; FU, *Fusobacterium ulcerans* ; FV, *Fusobacterium varium* .

Notably, the results showed that *F. nucleatum* was most prevalent in both cancer patients (regardless of cancer type and status) and controls, aligning with the population‐based findings from GGMP cohort (Figure 4A–E). Moreover, higher loads of *Fusobacterium*, especially *F. nucleatum* , *F. mortiferum* , *F. ulcerans* and *F. necrophorum* , were found in faeces of cancer patients compared to controls (Figure 4F–N). However, there was no significance for loads among four subspecies of *F. nucleatum* (Figure 4G–J). To explore the correlations of *Fusobacterium* to host conditions, correlation analyses were performed in both cancer patients and controls. Intriguingly, we discovered significant correlations between both *F. nucleatum* subsp. *polymorphum* and *F. mortiferum* and tumour types, both *F. nucleatum* subsp. *polymorphum* and *F. nucleatum* subsp. *nucleatum* and Hb, both *F. ulcerans* and *F. varium* and weight, both *F. nucleatum* subsp. *animals* and *F. mortiferum* and TRF (transferrin) in cancer patients (Figure 4O). *F. varium* was linked to height and fasting blood glucose, *F. ulcerans* to BMI and *F. necrophorum* to TRF (Figure 4O). Conversely, *F. ulcerans* showed a negative correlation with CEA (Figure 4O). As for controls, *F. nucleatum* subsp. *polymorphum* was linked to weight and BMI, *F. nucleatum* subsp. *vincentii* to triglyceride, *F. nucleatum* subsp. *animals* to CA199 (carbohydrate antigen 19–9) and *F. necrophorum* to fasting blood glucose and high‐density lipoprotein cholesterol (HDL) (Figure 4P). In the contract, *F. nucleatum* subsp. *vincentii* showed a negative correlation with HDL (Figure 4P). In addition, mixed infections were very common in both cancer patients and healthy controls (Figure S2A,B).

We further detected the levels of *Fusobacterium* species by the multiplex PCR assay, and examined their diagnostic performances in cancer. Interestingly, only *F. nucleatum, F. mortiferum*, *F. ulcerans* and *F. necrophorum* exhibited superior diagnostic performance, and *F. mortiferum* performed better than *F. nucleatum* , *F. ulcerans* and *F. necrophorum* in cancer diagnosis (AUROC 0.7109 vs. 0.5915, 0.5730 and 0.5692) (Figure S3A–D). While combinations of multiple bacterial markers increased the AUROC, they did not yield a statistically significant improvement over *F. mortiferum* alone. We then integrated the most promising bacterial markers with clinical parameters. Strikingly, a logistic regression model combining *F. nucleatum* , *F. mortiferum* , BMI, and Hb achieved a significantly higher diagnostic accuracy (AUROC = 0.9183) than any single bacterial marker (Figure 4Q, Figure S3E–H). This combination was identified as the optimal model, since the inclusion of additional *Fusobacterium* species (e.g., *F. ulcerans* and *F. necrophorum* ) did not further enhance the AUROC significantly (Table S13).

In addition, we explore the prevalence of *Fusobacterium* in cancer untreated, cancer treated and control group. The prevalence among them were 100%, 91.58% and 82.93%, respectively (Figure 4R). Although no obvious differences were observed in prevalence of *Fusobacterium* species (Figure 4S,T), the bacterial loads were significantly different between groups (Figure 4U–AC). Higher loads of *Fusobacterium*, especially *F. nucleatum* , *F. mortiferum* and *F. necrophorum* , were found in the faeces of cancer patients (untreated or treated) compared to controls (Figure 4U–AC). Intriguingly, the loads of *F. mortiferum* showed drastic increase in cancer group, but they were downregulated significantly after treatment (Figure 4Z).

### 3.5 Application of the Multiplex PCR Assay for Profiling *Fusobacterium* in Paired Tumour Tissues of CRC Patients and Lung Cancer Patients

The multiplex PCR assay was then employed to analyse the compositional features of *Fusobacterium* in colorectal tissue of CRC patients (21 patients, including 15 tumour tissues, 15 adjacent normal tissues and 16 normal tissues) (Figure S1C). Table S14 showed the characteristics of study participants. Compared to control group, the CRC group had higher age (69.50 (62.25–71.00), *p* < 0.001), higher SBP (systolic blood pressure) (136.50 (125.75–146.50), 0.028), higher positive rate of FOB (19 (90.48%), *p* < 0.001), higher CEA (5.10 (2.30–15.51), *p* < 0.001), lower BMI (23.04 (18.74–25.74), *p* = 0.048), lower UA (345.00 (298.00–404.75), *p* < 0.015), lower Hb (128.00 (113.50–143.00), *p* < 0.001) and lower ALT (14.00 (10.50–19.25), *p* < 0.001).

**FIGURE 5:**
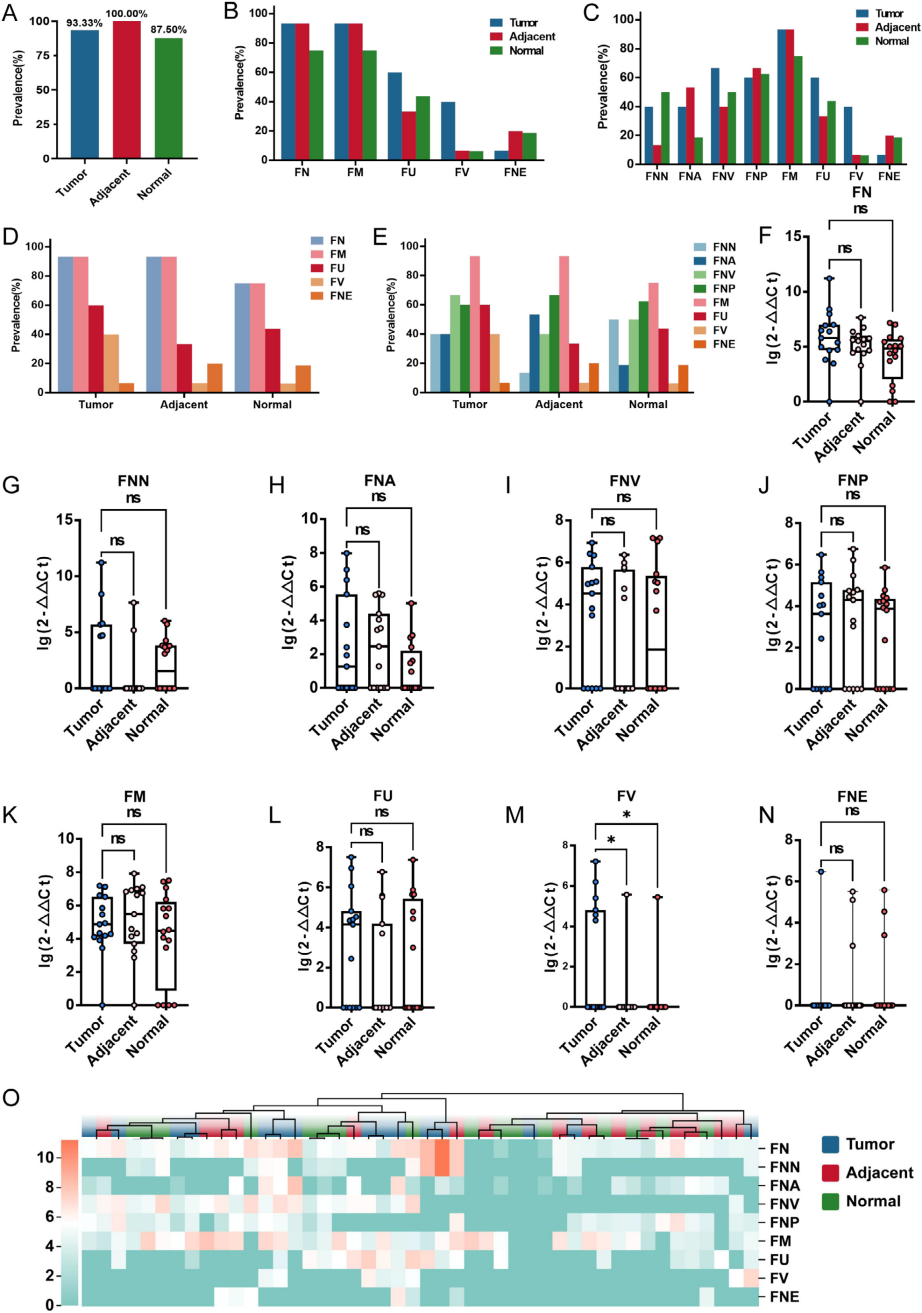
The compositional features of *Fusobacterium* communities in colorectal cancer tissues. (A) The positive detection rate of *Fusobacterium* in tumour tissues (*n* = 15), adjacent normal tissues (*n* = 15) and normal tissues (*n* = 16). (B‐E) The prevalence of *Fusobacterium* species and subspecies in tumour tissues (*n* = 15), adjacent normal tissues (*n* = 15) and normal tissues (*n* = 16). (F–N) The relative abundance of FN (F), FNN (G), FNA (H), FNV (I), FNP (J), FM (K), FU (L), FV (M) and FNE (N) in tumour (*n* = 15), adjacent normal (*n* = 15) and normal tissues (*n* = 16). Kruskal–Wallis H test followed by Dunn's test. (O) The detection of *Fusobacterium* in tumour (*n* = 15), adjacent normal (*n* = 15) and normal tissues (*n* = 15). Their relative quantitation in the *Fusobacterium* community of each sample are presented as a heatmap. The relative abundance of *Fusobacterium* was depicted using a colour scale, where red denoted high relative abundance and blue signified low relative abundance. FM, *Fusobacterium mortiferum* ; FN, *Fusobacterium nucleatum* ; FNA, *Fusobacterium nucleatum* subsp. *animalis*; FNE, *Fusobacterium necrophorum* .; FNN, *Fusobacterium nucleatum* subsp. *nucleatum*; FNP, *Fusobacterium nucleatum* subsp. *polymorphum*; FNV, *Fusobacterium nucleatum* subsp. *vincentii*; FU, *Fusobacterium ulcerans* ; FV, *Fusobacterium varium* .

In CRC patients, tumour tissues (93.33%) and adjacent normal tissues (100.00%) showed higher detection rate of *Fusobacterium* than normal tissues (87.50%) (Figure 5A). *F. nucleatum and F. mortiferum* were most prevalent among tumour, adjacent normal tissues and normal tissues, and except *F. necrophorum* , *F. nucleatum, F. mortiferum, F. ulcerans* and *F. varium* were more prevalent in tumour tissues than normal tissues (Figure 5B–E). At the subspecies level of *F. nucleatum* , only the detection rate of subsp. *animals* showed a clear downward trend between tumour tissues and normal tissues while other three subspecies did not vary like this (Figure 5C,E). Interestingly, higher loads of *F. varium* were found in tumour tissues compared to adjacent normal tissues and normal tissues (Figure 5M). However, there was no significance for loads among other *Fusobacterium* sp. (Figure 5F–L,N). Moreover, the coexistance of *Fusobacterium* species and subspecies were commonly detected in both tumour tissues and normal tissues, and the tumour tissues contained more species than normal tissues (Figure 5O and Figure S4). Especially, more mixed infections of *F. varium* were found in tumour tissues than in adjacent normal tissues and normal tissues (Figure S4).

Next, we explored the compositional features of *Fusobacterium* communities in the faeces and tumour tissues of lung cancer to reveal the correlations between *Fusobacterium* species and lung cancer. Here, 49 of lung cancer participants with 185 samples were analysed (Figure S1C). Table S14 showed the characteristics of study participants. The lung cancer group had higher age (57.00 (52.25–65.75), *p* < 0.001), higher SBP (126.50 (117.00–136.50), *p* = 0.014), higher CEA (2.70 (1.35–4.88), *p* < 0.001), lower UA (327.00 (276.25–383.25), *p* < 0.001) and lower Hb (128.00 (113.50–133.00), *p* < 0.001) compared to control group. The matched tumour, adjacent tissues, normal tissues, and faeces were employed to analyse the compositional features of *Fusobacterium* communities in these clinical samples.

**FIGURE 6:**
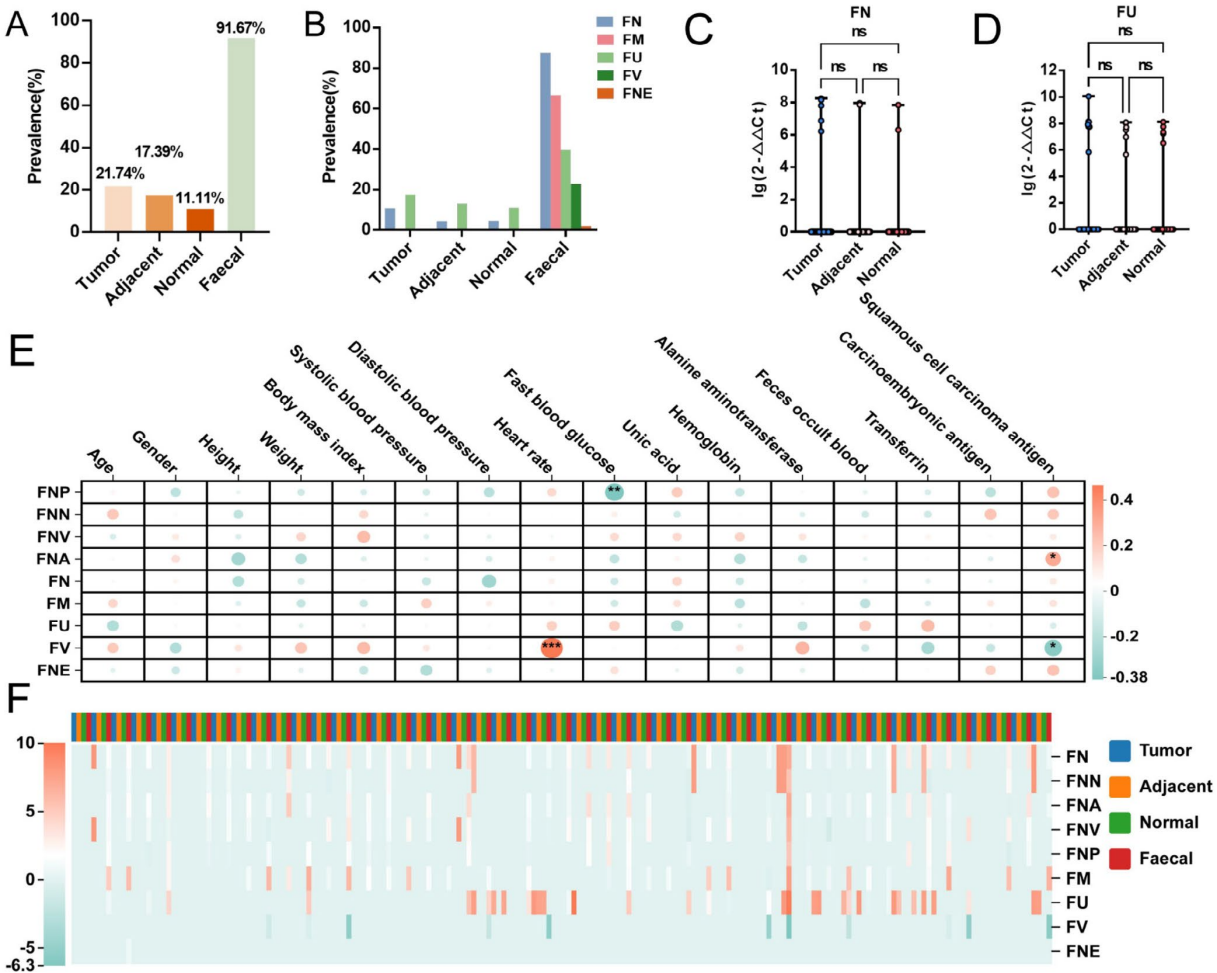
The identification of *Fusobacterium* in paired tumour tissues and faeces of lung cancer patients. (A, B) The prevalence of *Fusobacterium* (A) and *Fusobacterium* species (B) in paired tumour tissue (*n* = 46), adjacent normal tissue (*n* = 46), normal tissue (*n* = 45) and faeces (*n* = 48) of lung cancer patients. (C, D) The relative abundance of FN (C) and FU (D) in tumour tissue (*n* = 46), adjacent normal tissue (*n* = 46) and normal tissue (*n* = 45) of lung cancer patients. Kruskal–Wallis H test followed by Dunn's test. (E) The correlations between faecel *Fusobacterium* and host parameters (*n* = 48). The correlation analysis is spearman analysis. A higher absolute value of the correlation coefficient signifies a stronger correlation, with red denoting a positive correlation and blue indicating a negative correlation. The size of the nodes and the depth of their colour represent the strength of the correlation, where larger nodes and darker hues correspond to stronger correlations. An asterisk (*) denotes the *p*‐value. (F) The detection of *Fusobacterium* in tumour tissue (*n* = 46), adjacent normal tissue (*n* = 46) and normal tissue (*n* = 45) of lung cancer patients. Their relative quantitation in the *Fusobacterium* community of each sample are presented as a heatmap. The relative abundance of *Fusobacterium* was depicted using a colour scale, where red denoted high relative abundance and blue signified low relative abundance. FM, *Fusobacterium mortiferum* ; FN, *Fusobacterium nucleatum* ; FNA, *Fusobacterium nucleatum* subsp. *animalis*; FNE, *Fusobacterium necrophorum* .; FNN, *Fusobacterium nucleatum* subsp. *nucleatum*; FNP, *Fusobacterium nucleatum* subsp. *polymorphum*; FNV, *Fusobacterium nucleatum* subsp. *vincentii*; FU, *Fusobacterium ulcerans* ; FV, *Fusobacterium varium* .

Higher prevalence of *Fusobacterium* was found in the tumour tissues (21.74%) than in the adjacent normal tissues (17.39%) and in normal tissues (11.11%) (Figure 6A). Only *F. nucleatum* and *F. ulcerans* were identified in lung cancer (Figure 6B). While *F. nucleatum* and *F. ulcerans* gradually decreased in tumour tissues, adjacent normal tissues, and normal tissues, these changes did not reach statistical significance (Figure 6B–D). Correlations between *Fusobacterium* in faeces and host parameters were also analysed. Intriguingly, we discovered significant correlations between *F. varium* and HR (Figure 6E). *F. nucleatum* subsp. *animals* was linked to SCC (squamous cell carcinoma antigen) (Figure 6E). Conversely, *F. nucleatum* subsp. *polymorphum* showed a negative correlation with fasting blood glucose, while *F. varium* showed a negative correlation with SCC (Figure 6E). The prevalent features of *Fusobacterium* in faeces of lung cancer patients was similar to the cases of clinical cancer cohort, but there was no significant consistent correlation between lung cancer tissue and faeces in lung cancer patients (Figure 6A,B,F).

## 4 Discussion

Previous studies demonstrated conflicting results on prevalent features of *Fusobacterium* species in healthy or patient population. However, there is currently no efficient detection method to accurately determine *Fusobacterium* species in microbiota. To solve this critical problem, we developed a high‐precision modified method incorporating multiplex PCR assay that allows concurrent differentiation of five common species of *Fusobacterium* ( *F. nucleatum* , *F. mortiferum* , *F. ulcerans* , *F. varium* and *F. necrophorum* ) and four subspecies of *F. nucleatum* (*nucleatum*, *animalis*, *vincentii* and *polymorphum*). By employing the PCR method, we investigated the prevalent features of these common *Fusobacterium* species in Southern Chinese population and cancer patients. The results indicated that our new‐developed modified method incorporating multiplex PCR assay provides a more precise assessment of common *Fusobacterium* species in the context of both health and disease.

In the study, we uncovered that *F. nucleatum* was most prevalent in the Southern Chinese population regardless of disease status, which opposes previous findings by Chan et al. and He et al. that non‐nucleatum *Fusobacterium* is dominant in the Southern Chinese population (Yeoh et al. 2020; He et al. 2021). These previous studies indicate a high prevalence of non‐nucleatum *Fusobacterium* in the Southern Chinese population, linked to various host conditions (Yeoh et al. 2020; Geva‐Zatorsky et al. 2017; He et al. 2021). The prevalence and abundance of *Fusobacterium* lineages in Southern Chinese population are F3‐mortiferum (60.35% prevalence and 0.57% mean abundance), F2‐ulcerans/varium (20.36% and 0.11%), F1‐nucleatum (16.3% and 0.02%) and F4‐necrophorum (0.58% and 0.004%), respectively (He et al. 2021). However, the existing data on *Fusobacterium* within the population are derived from 16S rRNA gene sequencing. Due to the limitations of short‐read second‐generation sequencing, conventional 16S rRNA gene sequencing can only partially target variable regions (e.g., V3 and V4) within the 16S rRNA gene. These regions typically account for only about 30% of the total 16S rRNA gene length (approximately 1.5 kb), which compromises both the resolution and accuracy of bacterial identification. Consequently, standard 16S rRNA gene sequencing generally provides reliable genus‐level classification results while misses crucial species‐level information. It's well known that the 16S rRNA gene exhibits high similarity among *Fusobacterium* species, and consequently, it fails to offer species‐level resolution when utilised in 16S amplicon sequencing (Kim et al. 2010; Bi et al. 2022). However, PCR method is highly sensitive and specific for detecting *Fusobacterium* species since we designed specific primers and probes based on the specific gene marker of *Fusobacterium* species.

In addition to *F. nucleatum* , we revealed the prevalent features of other common *Fusobacterium* species and *F. nucleatum* subspecies in Southern Chinese population and cancer patients, and discovered the correlations to host conditions. Although *F. nucleatum, F. mortiferum* and *F. ulcerans* exhibited superior diagnostic performance for cancer, *F. mortiferum* performed better than *F. nucleatum* and *F. ulcerans* in cancer diagnosis, implying non‐nucleatum *Fusobacterium* species might have a differential role in the development of solid cancer. Moreover, we established a high‐performance diagnostic model by combining *F. nucleatum* , *F. mortiferum* , BMI and Hb. In addition to *Fusobacterium* prevalence, we revealed different change mode for *Fusobacterium* loads in faeces and tumour tissues of cancer patients both untreated and treated. This study may promote the understanding of how common *Fusobacterium* members promote solid cancer development and provide further directions to better model this malignant disease and study relevant host‐gut microbe interactions. Intriguingly, we found the low common *Fusobacterium* prevalence in lung cancer contrary to CRC. The reason for the low prevalence of *Fusobacterium* in lung tissues, in contrast to CRC, is most likely due to tissue tropism rather than technical limitations since we have rigorously evaluated the detection effect of our newly developed modified method incorporating multiplex PCR assay in both lung tissues and CRC samples. In the future, this may be a very worthwhile field to explore.

This study has some limitations. First, this study concentrates exclusively on the common *Fusobacterium* species prevalent among the population, and do not comprehensively elucidate the potential competitive or synergistic interactions between different *Fusobacterium* species, nor their clinical associations. Second, this study investigates the relatively small population. To expand the understanding of the distribution of *Fusobacterium* members in human and their associations with different cancer (especially CRC) as well as other diseases, large‐scale cohort studies on different populations with different disease statuses are required. Third, to adapt to the fact that most PCR detection systems on the market only have four fluorescence channels, our multiplex PCR assay was separated into three modified multiplex PCR assays. It is not an actual multiplex assay allowing simultaneous detection of these species/subspecies in one tube, but rather a modified multiplex qPCR assay. In the future, with the advancement of PCR detection technology, these primer sets will be integrated into one tube, and the actual multiplex PCR assay will be validated methodologically.

In conclusion, by large‐scale comparative genomic analysis, we successfully developed a high‐precision modified method incorporating multiplex PCR assay for the simultaneous identification of common *Fusobacterium* species and subspecies in both faeces and tumour tissues. The new‐developed modified method incorporating multiplex PCR technique will serve as a useful tool aiding future studies for common *Fusobacterium* in diseases. With the PCR technique, we provide a high‐resolution view of common *Fusobacterium* members in both normal populations and solid cancer patients. This study can help to clarify the association between common *Fusobacterium* and different solid cancers, and precisely probe the oncogenic culprit.

## Author Contributions

D.Z. and H.Z. conceived the project; T.S., J.L., X.L., X.X. and L.L. performed and analysed most experiments; Y.X., S.Z., B.L., S.Y., Y.Z., Z.Z., H.L. and S.C. performed and analysed experiments; T.S., H.L. and M.H. performed clinical investigation; T.S., J.L., X.L., L.Z. and D.Z. performed data visualisation; B.L., H.Z. and D.Z. provided funding support; D.Z., H.Z., C.W. and Z.L. supervised the experiments; T.S., C.W., H.Z. and D.Z. wrote the original draft; T.S., C.W., H.Z. and D.Z. performed review and editing.

## Funding

This work was supported by National Natural Science Foundation of China (82572635, 81925026, 82341218, 82130068 and 82402710); National Key Research and Development Program of China (2022YFA0806400); Guangdong Provincial Clinical Research Center for Laboratory Medicine (2023B110008); Science and Technology Program of Guangzhou (2025A04J3781); China Postdoctoral Science Foundation (2024M751324).

## Conflicts of Interest

The authors Hongwei Zhou, Dongxin Zhang, Xiaoxie Xu, Liqiong Li, Tingting Shen, Jiarui Liang and Xuyu Li have patent application related to this work.

## Supporting information


**Figures S1–S4:** mbt270292‐sup‐0001‐FigureS1‐S4.docx.

**Table S1:** mbt270292‐sup‐0002‐TableS1.xlsx.

**Table S2:** mbt270292‐sup‐0003‐TableS2.xlsx.

**Table S3:** mbt270292‐sup‐0004‐TableS3.xlsx.

**Table S4:** mbt270292‐sup‐0005‐TableS4.xlsx.

**Table S5:** mbt270292‐sup‐0006‐TableS5.xlsx.

**Table S6:** mbt270292‐sup‐0007‐TableS6.xlsx.

**Table S7:** mbt270292‐sup‐0008‐TableS7.xlsx.

**Table S8:** mbt270292‐sup‐0009‐TableS8.xlsx.

**Table S9:** mbt270292‐sup‐0010‐TableS9.xlsx.

**Table S10:** mbt270292‐sup‐0011‐TableS10.xlsx.

**Table S11:** mbt270292‐sup‐0012‐TableS11.xlsx.

**Table S12:** mbt270292‐sup‐0013‐TableS12.xlsx.

**Table S13:** mbt270292‐sup‐0014‐TableS13.xlsx.

**Table S14:** mbt270292‐sup‐0015‐TableS14.xlsx.

## Data Availability

All data are contained within the manuscript and Figures S1–S4 and Tables [Link], [Link]. The data that support the findings of this study are available from the corresponding author upon reasonable request.

## References

[mbt270292-bib-0001] Adriaans, B. , and H. Shah . 1988. “ *Fusobacterium ulcerans* Sp. Nov. From Tropical Ulcers.” International Journal of Systematic Bacteriology 38: 447–448.

[mbt270292-bib-0002] Alkharaan, H. , L. Lu , G. Gabarrini , et al. 2020. “Circulating and Salivary Antibodies to *Fusobacterium nucleatum* Are Associated With Cystic Pancreatic Neoplasm Malignancy.” Frontiers in Immunology 11: 2003. 32983143 10.3389/fimmu.2020.02003 PMC7484485

[mbt270292-bib-0004] Bi, D. , Y. Zhu , Y. Gao , et al. 2021. “A Newly Developed PCR‐Based Method Revealed Distinct *Fusobacterium nucleatum* Subspecies Infection Patterns in Colorectal Cancer.” Microbial Biotechnology 14: 2176–2186. 34309194 10.1111/1751-7915.13900 PMC8449656

[mbt270292-bib-0003] Bi, D. , Y. Zhu , Y. Gao , et al. 2022. “Profiling *Fusobacterium* Infection at High Taxonomic Resolution Reveals Lineage‐Specific Correlations in Colorectal cancer.” Nature Communications 13: 3336. 10.1038/s41467-022-30957-6 PMC9184491 35680952

[mbt270292-bib-0005] Brennan, C. A. , and W. S. Garrett . 2019. “ *Fusobacterium nucleatum* – Symbiont, Opportunist and Oncobacterium.” Nature Reviews. Microbiology 17: 156–166. 30546113 10.1038/s41579-018-0129-6 PMC6589823

[mbt270292-bib-0006] Bullman, S. , C. S. Pedamallu , E. Sicinska , et al. 2017. “Analysis of *Fusobacterium* Persistence and Antibiotic Response in Colorectal cancer.” Science 358: 1443–1448. 29170280 10.1126/science.aal5240 PMC5823247

[mbt270292-bib-0007] Castellarin, M. , R. L. Warren , J. D. Freeman , et al. 2012. “ *Fusobacterium nucleatum* Infection Is Prevalent in Human Colorectal Carcinoma.” Genome Research 22: 299–306. 22009989 10.1101/gr.126516.111 PMC3266037

[mbt270292-bib-0008] Citron, D. 2002. “Update on the Taxonomy and Clinical Aspects of the Genus *Fusobacterium* .” Clinical Infectious Diseases 35: S22–S27. 12173104 10.1086/341916

[mbt270292-bib-0009] Dai, Z. , O. O. Coker , G. Nakatsu , et al. 2018. “Multi‐Cohort Analysis of Colorectal Cancer Metagenome Identified Altered Bacteria Across Populations and Universal Bacterial Markers.” Microbiome 6: 70. 29642940 10.1186/s40168-018-0451-2 PMC5896039

[mbt270292-bib-0010] Gaiser, R. A. , A. Halimi , H. Alkharaan , et al. 2019. “Enrichment of Oral Microbiota in Early Cystic Precursors to Invasive Pancreatic Cancer.” Gut 68: 2186–2194. 30872392 10.1136/gutjnl-2018-317458 PMC6872446

[mbt270292-bib-0011] Geva‐Zatorsky, N. , E. Sefik , L. Kua , et al. 2017. “Mining the Human Gut Microbiota for Immunomodulatory Organisms.” Cell 168: 928–943.e11. 28215708 10.1016/j.cell.2017.01.022 PMC7774263

[mbt270292-bib-0012] He, Y. , P. Mujagond , W. Tang , et al. 2021. “Non‐Nucleatum *Fusobacterium* Species Are Dominant in the Southern Chinese Population With Distinctive Correlations to Host Diseases Compared With *F. nucleatum* .” Gut 70: 810–812. 32690601 10.1136/gutjnl-2020-322090

[mbt270292-bib-0013] He, Y. , W. Wu , S. Wu , et al. 2018. “Linking Gut Microbiota, Metabolic Syndrome and Economic Status Based on a Population‐Level Analysis.” Microbiome 6: 172. 30249275 10.1186/s40168-018-0557-6 PMC6154942

[mbt270292-bib-0014] He, Y. , W. Wu , H. M. Zheng , et al. 2018. “Regional Variation Limits Applications of Healthy Gut Microbiome Reference Ranges and Disease Models.” Nature Medicine 24: 1532–1535. 10.1038/s41591-018-0164-x 30150716

[mbt270292-bib-0015] Hieken, T. J. , J. Chen , T. L. Hoskin , et al. 2016. “The Microbiome of Aseptically Collected Human Breast Tissue in Benign and Malignant Disease.” Scientific Reports 6: 30751. 27485780 10.1038/srep30751 PMC4971513

[mbt270292-bib-0016] Hsieh, Y. Y. , S. Y. Tung , H. Y. Pan , et al. 2021. “ *Fusobacterium nucleatum* Colonization Is Associated With Decreased Survival of *helicobacter pylori* ‐Positive Gastric Cancer Patients.” World Journal of Gastroenterology 27: 7311–7323. 34876791 10.3748/wjg.v27.i42.7311 PMC8611209

[mbt270292-bib-0017] Kim, H. S. , D. S. Lee , Y. H. Chang , et al. 2010. “Application of rpoB and Zinc Protease Gene for Use in Molecular Discrimination of *Fusobacterium nucleatum* Subspecies.” Journal of Clinical Microbiology 48: 545–553. 19955278 10.1128/JCM.01631-09 PMC2815611

[mbt270292-bib-0018] Kostic, A. D. , E. Chun , L. Robertson , et al. 2013. “ *Fusobacterium nucleatum* Potentiates Intestinal Tumorigenesis and Modulates the Tumor‐Immune Microenvironment.” Cell Host & Microbe 14: 207–215. 23954159 10.1016/j.chom.2013.07.007 PMC3772512

[mbt270292-bib-0019] Krieger, M. , Y. M. AbdelRahman , D. Choi , et al. 2024. “Stratification of *Fusobacterium nucleatum* by Local Health Status in the Oral Cavity Defines Its Subspecies Disease Association.” Cell Host & Microbe 32, no. 4: 479–488.e4. 38479393 10.1016/j.chom.2024.02.010 PMC11018276

[mbt270292-bib-0020] Liu, D. , Y. Zhang , G. Fan , et al. 2022. “IPGA: A Handy Integrated Prokaryotes Genome and pan‐Genome Analysis Web Service.” iMeta 1: e55. 38867900 10.1002/imt2.55 PMC10989949

[mbt270292-bib-0021] Manson McGuire, A. , K. Cochrane , A. D. Griggs , et al. 2014. “Evolution of Invasion in a Diverse Set of *Fusobacterium* Species.” MBio 5: e01864. 25370491 10.1128/mBio.01864-14 PMC4222103

[mbt270292-bib-0022] Nakatsu, G. , X. Li , H. Zhou , et al. 2015. “Gut Mucosal Microbiome Across Stages of Colorectal Carcinogenesis.” Nature Communications 6: 8727. 10.1038/ncomms9727 PMC4640069 26515465

[mbt270292-bib-0023] Nejman, D. , I. Livyatan , G. Fuks , et al. 2020. “The Human Tumor Microbiome Is Composed of Tumor Type‐Specific Intracellular Bacteria.” Science 368: 973–980. 32467386 10.1126/science.aay9189 PMC7757858

[mbt270292-bib-0024] Ohkusa, T. , I. Okayasu , T. Ogihara , K. Morita , M. Ogawa , and N. Sato . 2003. “Induction of Experimental Ulcerative Colitis by *Fusobacterium varium* Isolated From Colonic Mucosa of Patients With Ulcerative Colitis.” Gut 52: 79–83. 12477765 10.1136/gut.52.1.79 PMC1773498

[mbt270292-bib-0025] Ohkusa, T. , N. Sato , T. Ogihara , K. Morita , M. Ogawa , and I. Okayasu . 2002. “ *Fusobacterium varium* Localized in the Colonic Mucosa of Patients With Ulcerative Colitis Stimulates Species‐Specific Antibody.” Journal of Gastroenterology and Hepatology 17: 849–853. 12164960 10.1046/j.1440-1746.2002.02834.x

[mbt270292-bib-0026] Riordan, T. 2007. “Human Infection With *Fusobacterium necrophorum* (Necrobacillosis), with a Focus on Lemierre's Syndrome.” Clinical Microbiology Reviews 20: 622–659. 17934077 10.1128/CMR.00011-07 PMC2176048

[mbt270292-bib-0027] Strauss, J. , A. White , C. Ambrose , J. McDonald , and E. Allen‐Vercoe . 2008. “Phenotypic and Genotypic Analyses of Clinical *Fusobacterium nucleatum* and *Fusobacterium periodonticum* Isolates From the Human Gut.” Anaerobe 14: 301–309. 19114111 10.1016/j.anaerobe.2008.12.003

[mbt270292-bib-0028] Thomas, A. M. , P. Manghi , F. Asnicar , et al. 2019. “Metagenomic Analysis of Colorectal Cancer Datasets Identifies Cross‐Cohort Microbial Diagnostic Signatures and a Link With Choline Degradation.” Nature Medicine 25: 667–678. 10.1038/s41591-019-0405-7 PMC9533319 30936548

[mbt270292-bib-0029] Wang, N. , and J. Y. Fang . 2023. “ *Fusobacterium nucleatum* , A Key Pathogenic Factor and Microbial Biomarker for Colorectal cancer.” Trends in Microbiology 31: 159–172. 36058786 10.1016/j.tim.2022.08.010

[mbt270292-bib-0030] Wirbel, J. , P. T. Pyl , E. Kartal , et al. 2019. “Meta‐Analysis of Fecal Metagenomes Reveals Global Microbial Signatures That Are Specific for Colorectal cancer.” Nature Medicine 25: 679–689. 10.1038/s41591-019-0406-6 PMC7984229 30936547

[mbt270292-bib-0031] Yamamura, K. , Y. Baba , S. Nakagawa , et al. 2016. “Human Microbiome *Fusobacterium nucleatum* in Esophageal Cancer Tissue Is Associated With Prognosis.” Clinical Cancer Research 22: 5574–5581. 27769987 10.1158/1078-0432.CCR-16-1786

[mbt270292-bib-0032] Yamamura, K. , D. Izumi , R. Kandimalla , et al. 2019. “Intratumoral *Fusobacterium nucleatum* Levels Predict Therapeutic Response to Neoadjuvant Vhemotherapy in Esophageal Squamous Cell Carcinoma.” Clinical Cancer Research 25: 6170–6179. 31358543 10.1158/1078-0432.CCR-19-0318 PMC6801075

[mbt270292-bib-0033] Yang, S. , S. Lin , G. D. Kelen , et al. 2002. “Quantitative Multiprobe PCR Assay for Simultaneous Detection and Identification to Species Level of Bacterial Pathogens.” Journal of Clinical Microbiology 40: 3449–3454. 12202592 10.1128/JCM.40.9.3449-3454.2002 PMC130696

[mbt270292-bib-0034] Yeoh, Y. K. , Z. Chen , M. C. S. Wong , et al. 2020. “Southern Chinese Populations Harbour Non‐Nucleatum Fusobacteria Possessing Homologues of the Colorectal Cancer‐Associated FadA Virulence Factor.” Gut 69: 1998–2007. 32051205 10.1136/gutjnl-2019-319635 PMC7569397

[mbt270292-bib-0035] Yu, J. , Q. Feng , S. H. Wong , et al. 2017. “Metagenomic Analysis of Faecal Microbiome as a Tool Towards Targeted Non‐Invasive Biomarkers for Colorectal Cancer.” Gut 66: 70–78. 26408641 10.1136/gutjnl-2015-309800

