# Supplementary material for: A Modified Method Incorporating Multiplex PCR Reveals Fusobacterium Prevalence in Southern Chinese Population and Its Correlations in Cancers
Source: Microb Biotechnol. 2025 Dec 25;18(12):e70292. doi: 10.1111/1751-7915.70292 (PMC12740677; doi:10.1111/1751-7915.70292)
Supplement: Supplementary file 1 — Figures S1–S4: mbt270292‐sup‐0001‐FigureS1‐S4.docx. [file MBT2-18-e70292-s011.docx]

**
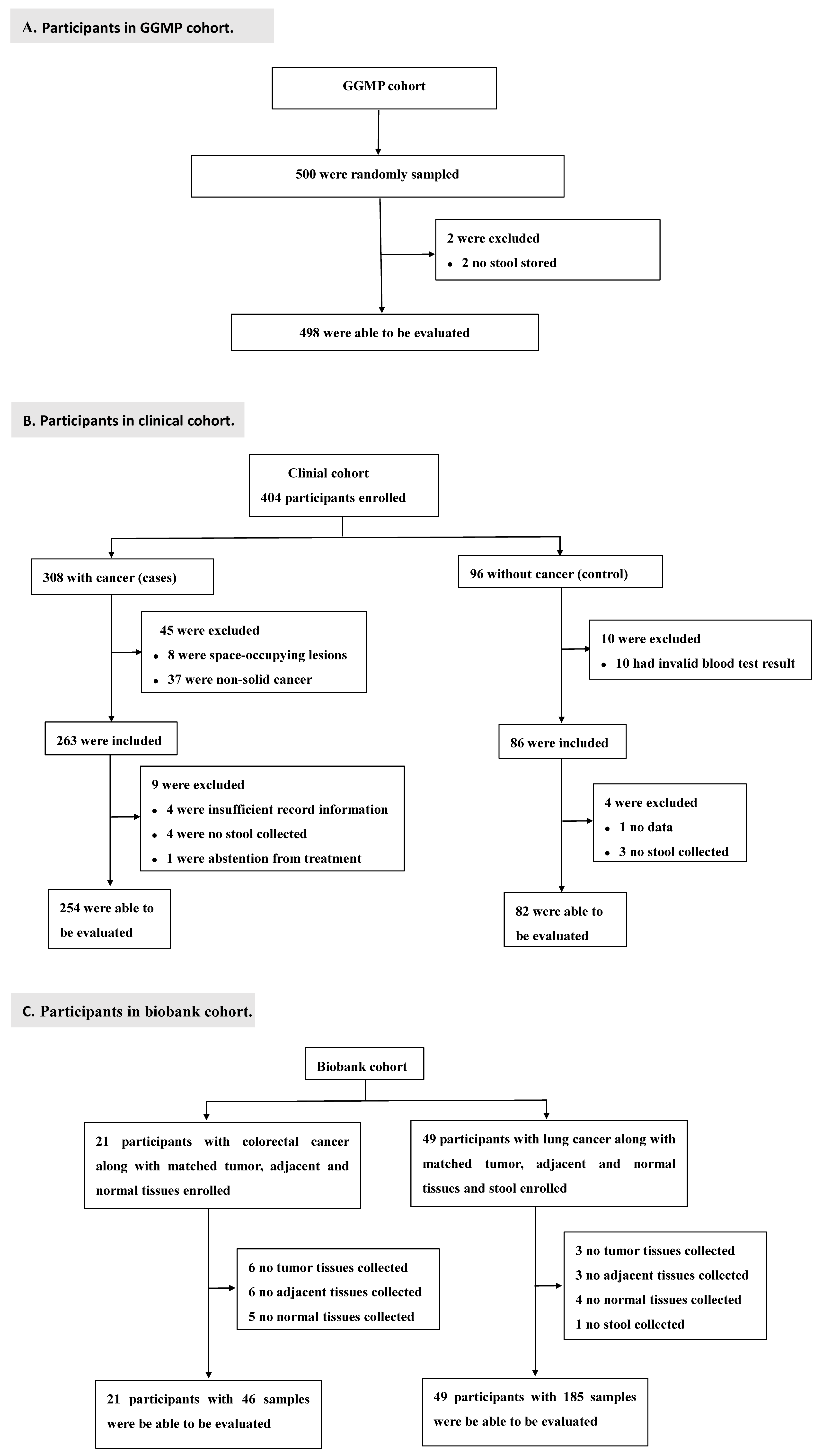
**

**Fig. S1 Human study subjects and sample collection.** A total of 904 participants were enrolled. (A) Participants in the GGMP cohort. (B) Participants in the clinical cohort. (C) Participants in the biobank cohort.


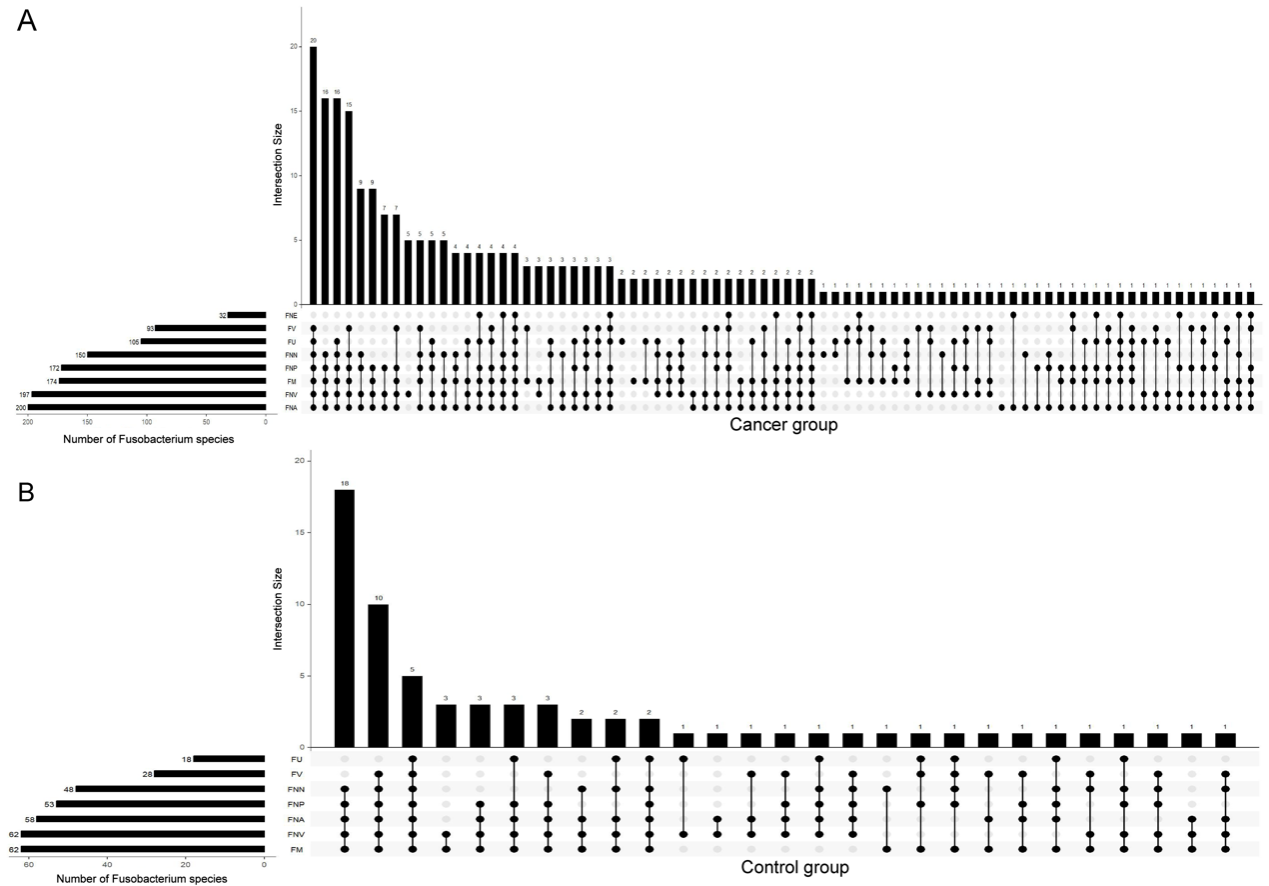


**Fig. S2 The infection patterns** **of *Fusobacterium* in faeces of cancer patients and normal controls.** The infection patterns of *Fusobacterium* species in faeces of cancer group (n=254) (A) and control group (n=82) (B).


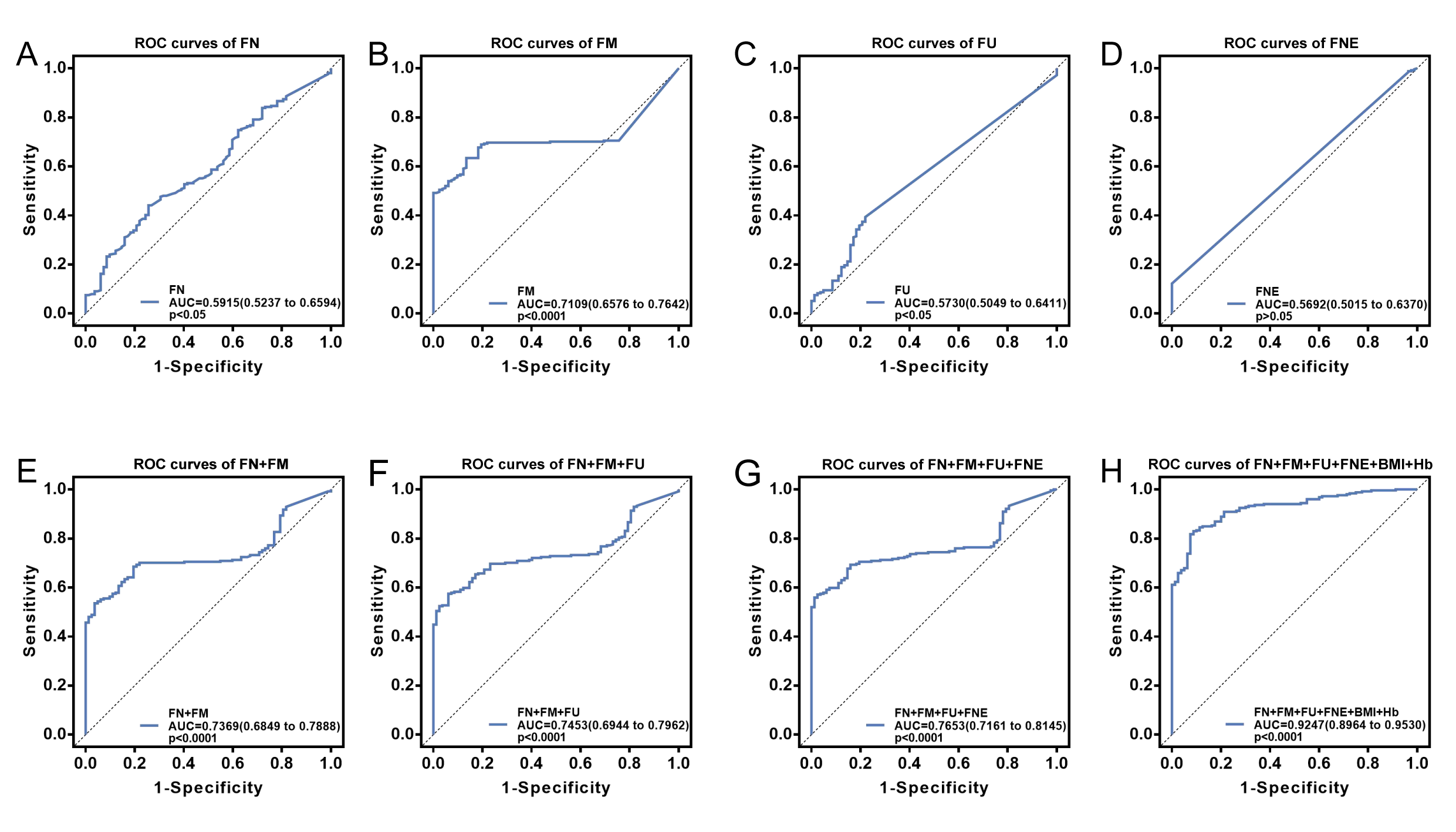


**Fig. S3 The receiver operating characteristic (ROC) curves of *Fusobacterium* species for predicting cancers.** (A) ROC curve of FN; (B) ROC curve of FM; (C) ROC curve of FU; (D) ROC curve of FNE; (E) ROC curve of combination of FN and FM; (F) ROC curve of combination of FN, FM, and FU; (G) ROC curve of combination of FN, FM, FU, and FNE; (H) ROC curve of combination of FN, FM, FU, and FNE with BMI and Hb. AUC, the area under the ROC curve. The 95% confidence intervals are shown in brackets. FN, *Fusobacterium nucleatum*; FM, *Fusobacterium mortiferum*; FU, *Fusobacterium ulcerans*; FNE, *Fusobacterium necrophorum*. BMI, body mass index; Hb, hemoglobin.


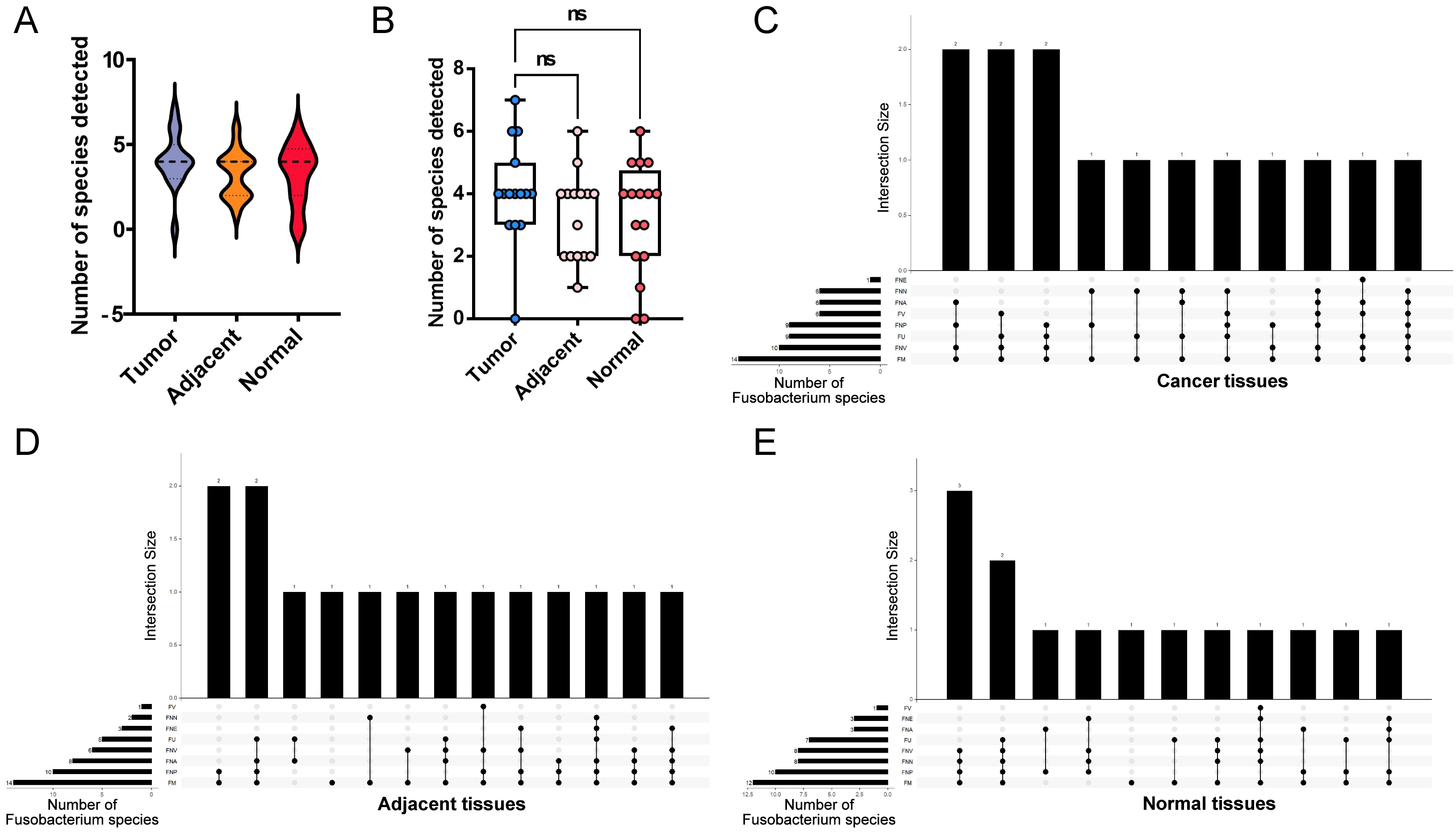


**Fig. S4 The infection patterns** **of *Fusobacterium* in CRC tissues.** (A-B) Numbers of *Fusobacterium* species detected in tumor tissues (n=15), adjacent normal tissues (n=15) and normal tissues (n=16). Kruskal-Wallis H test followed by Dunn’s test. (C-E) The infection patterns among different *Fusobacterium* species in tumor tissues (n=15) (C), adjacent normal tissues (n=15) (D) and normal tissues (n=16) (E).
